# Chalcogenido‐Dimethylgallates and ‐Indates DMPyr_2_[Me_2_M(μ_2_−E)]_2_ (M=Ga, In; E=S, Se): Building Blocks for Higher and Lower Order Chalcogenidoindates

**DOI:** 10.1002/open.202000347

**Published:** 2021-02-10

**Authors:** Jannick Guschlbauer, Tobias Vollgraff, Lars H. Finger, Klaus Harms, Jörg Sundermeyer

**Affiliations:** ^1^ Fachbereich Chemie and Materials Science Center Philipps-Universität Hans-Meerwein-Str. 4 35032 Marburg Germany

**Keywords:** Ionic liquids, chalcogenido metalates, chalcogenide materials, gallium, indium

## Abstract

Metalation of the anions in the ionic liquids DMPyr[SH] and DMPyr[SeH] (DMPyr=1,1‐dimethylpyrrolidinium) by trimethylgallium and trimethylindium is investigated. The reaction proceeds via pre‐coordination of [EH]^−^, methane elimination and formation of an unprecedented series of chalcogenido metalates DMPyr_2_[Me_2_M(*μ_2_*−E)]_2_ (M=Ga, In; E=S, Se). These show the presences of dinuclear dianions with four‐membered ring structures displaying highly nucleophilic bridging chalcogenide ligands in their crystallographically determined molecular structures. Some representative reactions of these building blocks with amphoteric electrophiles were studied: Addition of two equivalents of E(SiMe_3_)_2_ (E=S, Se) to the indates DMPyr_2_[Me_2_In(*μ*_2_−S)]_2_ and DMPyr_2_[Me_2_In(*μ_2_*−Se)]_2_ leads to a cleavage of the ring, E silylation and formation of mononuclear, monoanionic indates DMPyr[Me_2_In(SSiMe_3_)_2_], DMPyr[Me_2_In(SeSiMe_3_)_2_], and even a mixed sulfido‐selenido dimethylindate DMPyr[Me_2_In(SSiMe_3_)(SeSiMe_3_)]. Reaction of DMPyr_2_[Me_2_In(μ_2_−S)]_2_ with two equivalents of Lewis acid Me_3_In leads to charge delocalization, ring expansion and formation of six‐membered ring DMPyr_3_[Me_2_In(*μ*_2_−S−InMe_3_)]_3_. The latter is a key intermediate in the formation of dianionic sulfidoindate DMPyr_2_[(Me_2_In)_6_(*μ*_3_−S)_4_] displaying an unusual inverse heteroadamantane cage structure with four capping sulfido ligands.

## Graphical abstract

**Thermodynamics in action**! Condensation of Me_3_Ga and Me_3_In with anions [EH]^−^ (E=S, Se) to four‐membered rings, their cleavage or expansion and condensation to a cluster with inverse adamantane structure is driven by methane elimination, charge delocalization and dissociation.

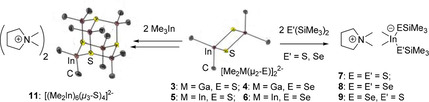

## 1 Introduction

Recently, we reported an atom economic synthesis of analytically pure ionic liquids and organic cation salts comprising hydrochalcogenide anions [SH]^−^, [SeH]^−^, [TeH]^−^.[^1^, ^2^, ^3^] Readily available methycarbonate ionic liquids Cat[OCO_2_Me] served as starting materials. We became interested to investigate the deprotonation and metalation of hydrosulfide and hydroselenide anions by trimethylgallium and ‐indium. Metal organic molecular compounds incorporating group 13 and 16 elements are of interest due to their potential to act as thermally labile precursors for triel‐chalcogenide‐based III–VI semiconductor materials.^[4]^ Particular interest is focussing on binary chalcogenides such as 2D‐GaSe, a photoconductor applied in non‐linear optics for frequency doubling^[5]^ or sesquiselenide In_2_Se_3_,^[6]^ but also on molecular precursors^[7]^ for ternary and quaternary members of the CIGS family of materials Cu(In_*x*_Ga_1−*x*_)(S_*y*_Se_1−*y*_)_2_ with their remarkable performance in optoelectronic^[6]^ and photovoltaic^[8]^ devices, respectively.

**Scheme 1:**
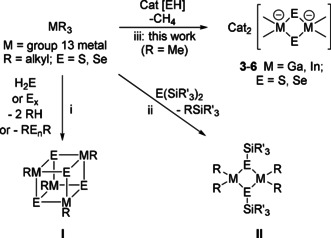
Syntheses of group 13/16 metal chalcogenido compounds: i: heterocubanes [RM(*μ*_3_−E)] (I)[^13^, ^14^] ii: silylchalcogenide dimers [R_2_M(*μ_2_*−ESiR_3_)]_2_ (II)[^15^, ^16^] and iii: new chalcogenido‐organometalates 3–6.

Common strategies to incorporate purely inorganic sulfur and selenium ions into molecular precursor compounds or clusters involve deprotonation of H_2_E (E=S, Se) with metal compounds containing basic leaving groups such as alkyl or amido ligands or salt elimination reactions of alkali metal chalcogenides M_2_E, M[EH], Li[ESiMe_3_]^[9]^ or Na[ESiMe_3_] with group 13 metal halides.[^10^, ^11^] Trimethylgallium and ‐indium are widely used group 13 precursors, which can be thermally decomposed to yield high purity semiconductor materials in MOCVD processes.^[12]^ Alkyltrieles are Lewis‐acids but can also act as Brønsted bases or nucleophiles towards H_2_E, E_8_ and (Me_3_Si)_2_E (E=S, Se). Scheme 1 displays representative reaction patterns of organotriels with such chalcogen sources.

When H_2_E or elemental chalcogens are reacted with trialkyltrieles, heterocubane compounds [(*μ*_3_−E)_4_(MR)_4_] (M=Al, Ga, In; R=Me_2_EtC, *t*Bu) emerge (Scheme 1,i).[^13^, ^14^] By deprotonation of mercapto silanes HSSiR’_3_ (R’=alkyl), or by desilylation of bistrialkylsilylchalcogenides with trialkyltrieles, the formation of dinuclear neutral complexes with bridging silylchalcogenido ligands [R_2_M(*μ_2_*−ESiR’_3_)]_2_ was observed (Scheme 1,ii).[^15^, ^16^] The availability of highly pure, water and chloride free organic cation salts Cat[SH] and Cat[SeH] inspired us to follow up a new strategy for synthesizing anionic chalcogenido metalates via metalation and protolysis of Cat[EH] by Me_3_M (Ga, In). The primary products **3**–**6** of this investigation are displayed in Scheme 1,iii.

Very few examples of such lower nuclearity chalcogenido gallates and indates with purely inorganic chalcogenido ligands are known such as the trinuclear six‐membered ring compounds [(Cl_2_M)(*μ_2_*−E)]_3_^3−^ (M=Ga, In; E=S, Se).^[17]^ They were obtained by reaction of alkali metal hydrochalcogenides with MCl_3_ and characterized after cation exchange for quaternary ammonium cations. It seems, that their existence requires a highly negative, trianionic charge in order to inhibit the formation of higher nuclear clusters.

## 2 Results and discussion

### 2.1 Synthesis and Characterisation of the Chalcogenide IL Building Blocks

**Scheme 2:**
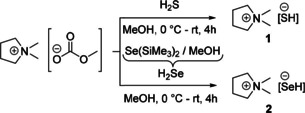
Preparation of DMPyr[SH] (1) and DMPyr[SeH] (2).

First, the synthesis of better crystallizing symmetric 1,1‐dimethylpyrrolidinium (DMPyr) hydrochalcogenide salts was performed following our protocol described for corresponding 1‐butyl‐1‐methylpyrrolidinium (BMPyr) salts (Scheme 2).^[2]^ DMPyr[SH] (**1**) was prepared by deprotonation of H_2_S with DMPyr[OCO_2_Me] in methanol. DMPyr[SeH] (**2**) was prepared by adding Se(SiMe_3_)_2_ to a solution of DMPyr[OCO_2_Me] in methanol.

**Figure 1:**
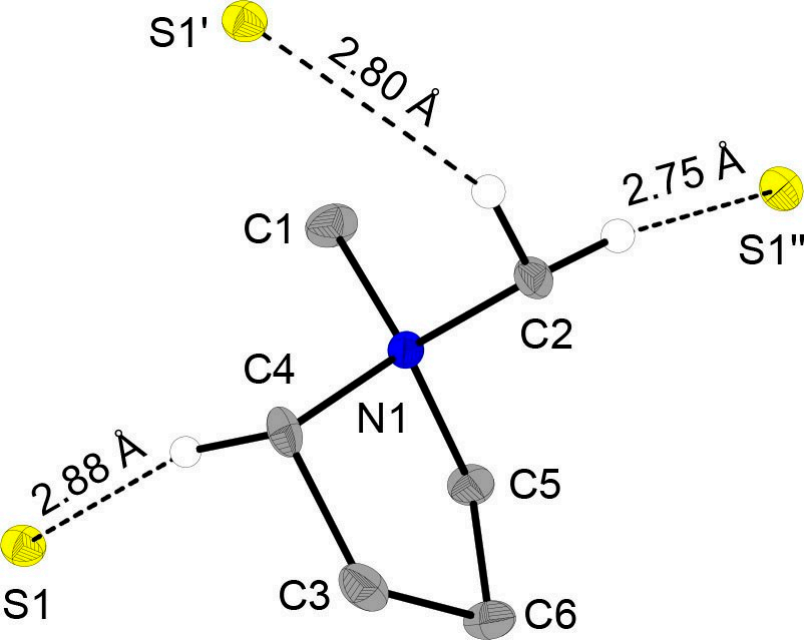
Molecular structure of DMPyr[SH] (**1**). Only H‐atoms involved in H‐bonds are shown. Ellipsoids shown at the 50 % level. Symmetry operations I: −1+x, y, z; II: −1/2+x, 1/2 −y, 1/2+z. Selected bond lengths (in Å) and angles (in °): C1−N1 1.493(2), C2−N1 1.503(1), C4−N1 1.511(1), C5−N1 1.511(1), C4−C3 1.527(2), C3−C6 1.550(2), C5−C6 1.526(2), C1−N1−C2 109.95(8), C1−N1−C5 111.67(8), C1−N1−C5−C6−161.66(9), C5−C6−C3 104.96(9), C5−C6−C3−C4−2.2(1), C6−C3−C4 105.59(9), C6−C3−C4−N1−23.7(1), C3−C4−N1−C2−77.3(1), S1−C4 3.776(1), S1−C4−N1 94.00(6), S1−C4−N1−C5−64.6(7).

Single crystals of DMPyr[SH] (**1**) suitable for X‐ray analysis were obtained by slow diffusion of diethyl ether into a solution of **1** in acetonitrile at room temperature. **1** crystallizes in space group *P*2_1_/*n* with four formula units per unit cell. In the lattice, one DMPyr^+^ cation is non‐covalently bonded to three hydrosulfide anions via H‐bonds ranging from 2.75 Å to 2.88 Å (Figure 1). Crystalline BMPyr[SH] does show exactly the same coordination pattern of the cation to three sulfur atoms and H‐bonds ranging from 2.64 Å to 2.87 Å.^[2]^

**Figure 2:**
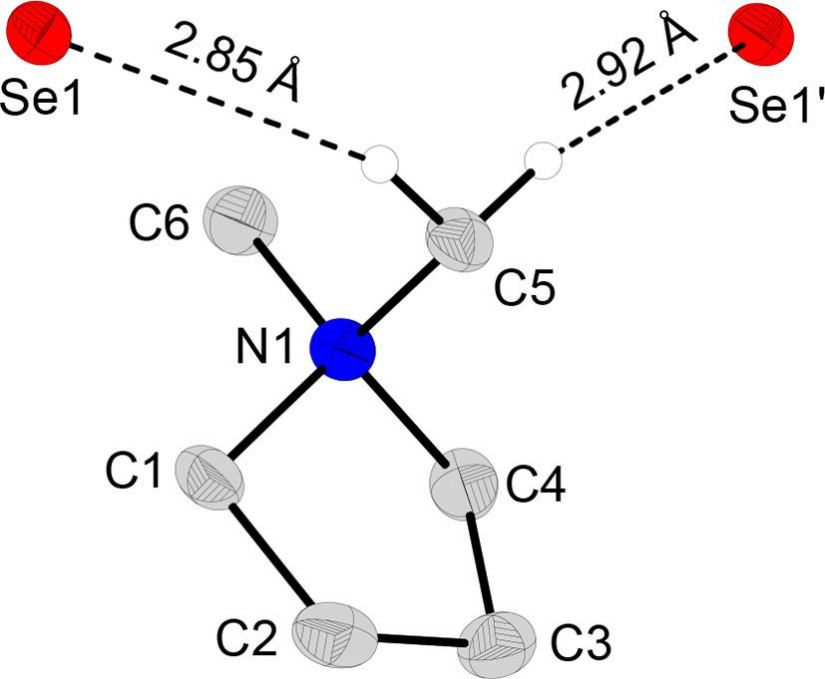
Molecular structure of DMPyr[SeH] (**2**). Only H‐atoms involved in H‐bonds are shown. Ellipsoids shown at the 50 % level. Symmetry operations I: 1/2+x, 1/2 −y, 1/2+z. Selected bond lengths (in Å) and angles (in °): C6−N1 1.495(4), C5−N1 1.497(4), C1−N1 1.511(4), C4−N1 1.513(4), C1−C2 1.529(5), C2−C3 1.546(5), C4−C3 1.528(5), C6−N1−C5 109.9(2), C6−N1−C4 111.3(2), C6−N1−C4−C3−159.4(3), C4−C3−C2 105.8(2), C4−C3−C2−C1 3.9(3), C3−C2−C1 104.9(3), C3−C2−C1−N1−28.7(3), C2−C1−N1−C5−75.0(3), Se1−C5 3.758(3), Se1−C5−N1 92.8(2), Se1−C5−N1−C4−170.6(2).

X‐ray diffractive single‐crystals of DMPyr[SeH] (**2**) were obtained by slow gas phase diffusion of diethyl ether into a saturated solution of **2** in a mixture of acetonitrile and diethyl ether. DMPyr[SeH] (**2**) crystallizes in space group *P*2_1_/*n* with four ion pairs per unit cell. One DMPyr^+^ cation is non‐covalently bonded to two selenium atoms by two H‐bonds to two protons of one and the same *N*‐methyl group ranging from 2.85 Å to 2.92 Å (Figure 2).

The closest interionic contacts of non‐cyclic quaternary ammonium salts R_4_N[SeH] (R=Me,^[20]^ (3.12 Å) and Bu^[19]^ (3.06 Å) are longer, while those of imidazolium salt EMIm[SeH]^[2]^ are in a similar short range 2.81 Å to 2.95 Å as observed in **2**.

**Figure 3:**
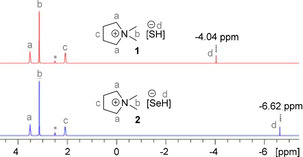
^1^H NMR (300.3 MHz, *dmso‐d_6_) of DMPyr[SH] (**1**) and DMPyr[SeH] (**2**).

The ^1^H NMR spectra of the salts **1** and **2** confirm previously reported proton shifts of the weakly solvated anions [SH]^−^ (−4.04 ppm) in **1** and [SeH]^−^ (−6.62 ppm) in **2**.^[2]^ The distinctive proton NMR spectra are presented in Figure 3.

### 2.2 Synthesis and Characterisation of the Chalcogenido Metalate Building Blocks

**Scheme 3:**
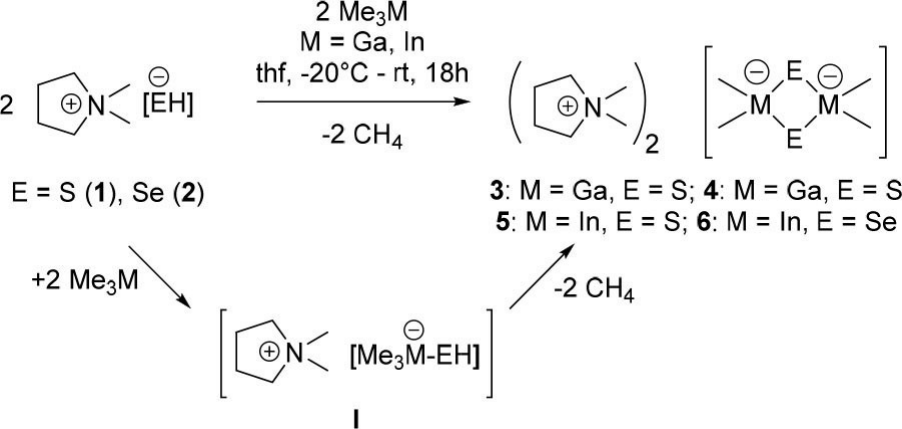
Preparation 3–6 from 1 and 2 via plausible intermediates I.

When a solution of Me_3_M (M=Ga, In) in THF is added to a THF suspension of DMPyr[EH] (E=S, Se) at −20 C, a clear solution is obtained within a few minutes. This might be explained by the formation of lipophilic intermediates DMPyr[Me_3_M−EH] (M=Ga, In; E=S, Se) (Scheme 3,I). After warming this solution to room temperature, a colourless precipitate emerges within few hours. ^1^H NMR spectra and elemental analyses of these isolated precipitates confirm the presence of the dianionic title compounds DMPyr_2_[Me_2_M(*μ_2_*−E)]_2_ **3**–**6** formed via methane elimination. Single‐crystal X‐ray analyses prove the presence of four‐membered ring structures of dianions [Me_2_M(*μ_2_*−E)]_2_^2−^ for all four combinations of E=S, Se and M=Ga, In (Scheme 3).

**Figure 4:**
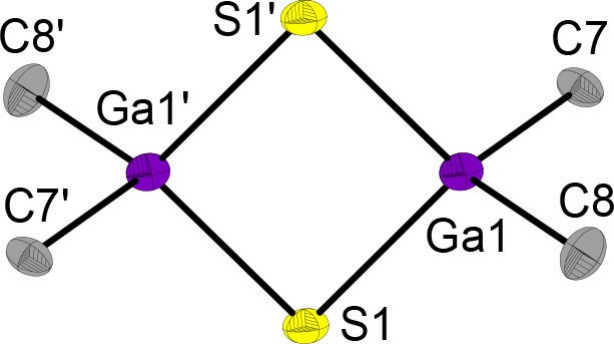
Molecular structure of the dianion present in DMPyr_2_[Me_2_Ga(*μ_2_*−S)]_2_ (**3**). Cations and H‐atoms are not shown for clarity. Due to disordered cations, non‐covalent interionic interactions cannot be discussed. Ellipsoids shown at the 50 % level. Symmetry operations I: 1−x, −y, 1−z. Selected bond lengths (in Å) and angles (in °) of the anion: Ga1−S1 2.3225(5), Ga1−S1’ 2.3293(4), S1−Ga1−S1’ 97.27(1), Ga1−S1’−Ga1’ 82.73(1), Ga1−C7 2.032(2), Ga1−C8 2.012(2), C7−Ga1−C8 108.62(7), Ga1’−S1’−Ga1−C7 117.38(5), Ga1’−S1−Ga1−C8 119.00(5).

Single crystals of DMPyr_2_[Me_2_Ga(*μ_2_*−S)]_2_ (**3**) were obtained by diffusion of pentane into a saturated solution of **3** in tetrahydrofuran at room temperature. **3** crystallizes in the monoclinic space group *P*2_1_/*n* with two ion pairs per unit cell. Due to crystallographically disordered cations the interionic interactions could not be reliably identified in this particular case (Figure 4).

**Figure 5:**
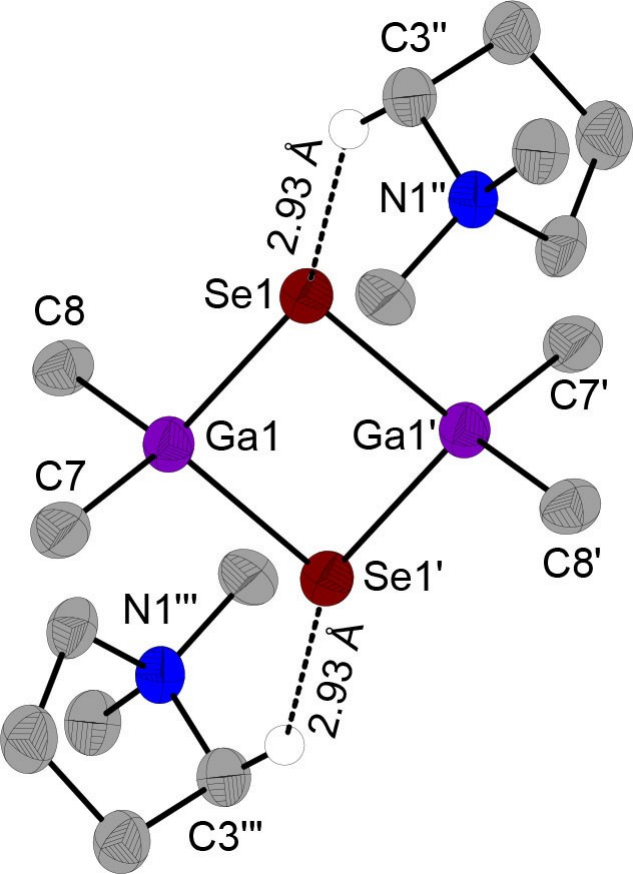
Molecular structure of DMPyr_2_[Me_2_Ga(*μ_2_*−Se)]_2_ (**4**). Only H‐atoms active in H‐bonds shown. Ellipsoids shown at the 50 % level. Symmetry operations I: 1−x, 1−y, 1−z; II: −1/2+x, 1/2‐y, 1/2 +z; III: 3/2−x, 1/2+y, 1/2−z. Selected bond lengths (in Å) and angles (in °) of the anion: Ga1−Se1 2.453(1), Ga1−Se1’ 2.461(1), Se1−Ga1−Se1’ 82.41(4), Ga1−Se1−Ga1’ 82.59(4), Ga1−C7 2.017(9), Ga1−C8 2.014(9), C7−Ga1−C8 109.0(4), Ga1’−Se1’−Ga1−C7−118.4(3), Ga1’−Se1−Ga1−C8−117.6(3).

Single crystals of DMPyr_2_[Me_2_Ga(*μ_2_*−Se)]_2_ (**4**) were obtained by diffusion of pentane into a saturated solution of **4** in tetrahydrofuran at 0 °C. **4** crystallizes in the monoclinic space group *P*2_1_/*n* with two ion pairs per unit cell. The compound displays a crystallographic center of symmetry. One crystallographically unique interionic H‐bond of 2.93 Å is identified as shortest anion‐cation contact (Figure 5).

**Figure 6:**
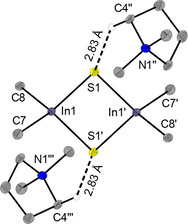
Molecular structure of DMPyr_2_[Me_2_In(*μ_2_*−S)]_2_ (**5**). Only H‐atoms active in H‐bonds are shown. Ellipsoids shown at the 50 % level. Symmetry operations I: −x, −y, −z; II: 1−x, −y, −z; III: −1+x, y, z. Selected bond lengths (in Å) and angles (in °) of the anion: In1−S1 2.5040(5), In1−S1’ 2.5067(4), S1−In1−S1’ 96.15(1), In1−S1−In1’ 83.85(1), In1−C7 2.206(2), In1−C8 2.205(2), C7−In1−C8 108.45(6), In1’−S1’−In1‐C7−118.81(5), In1’−S1−In1−C8 116.64(4).

Single crystals of DMPyr_2_[Me_2_In(*μ_2_*−S)]_2_ (**5**) obtained by diffusion of pentane into a saturated solution of **5** in tetrahydrofuran at room. **5** crystallizes in the monoclinic space group *P*2_1_/*n* with two ion pairs per unit cell. The non‐covalent interactions show remarkable similarity to those identified in the case of the selenium/gallium homologue **4**. Again, one crystallographically unique interionic H‐bond (2.83 Å) between one sulfur atom and the same ring C−H bond of the cation DMPyr^+^ is identified (Figure 6).

**Figure 7:**
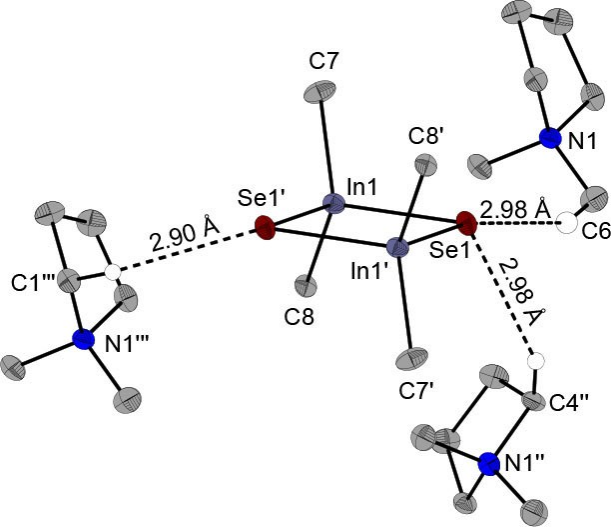
Molecular structure of DMPyr_2_[Me_2_In(*μ_2_*−Se)]_2_ (**6**). Only H‐atoms active in crystallographically unique H‐bonds are shown. Ellipsoids shown at the 50 % level. Symmetry operations I: 2−x, −y, 2−z; II: 1/2+x, 1/2 −y, 1/2+z; III: 1+x, y, z. Selected bond lengths (in Å) and angles (in °) of the anion: In1−Se1 2.6208(4), In1−Se1’ 2.6177(4), Se1−In1−Se1’ 96.72(1), In1−Se1−In1’ 83.28(1), In1−C7 2.209(3), In1−C8 2.212(3), C7−In1−C8 110.2(1), In1’−Se1’−In1−C7−117.35(9), In1’−Se1−In1−C8−116.58(8).

While DMPyr_2_[Me_2_In(*μ_2_*−Se)]_2_ (**6**) can be synthesized according to Scheme 3, single crystals suitable for X‐ray analysis were obtained by a different synthesis route: Leaving a solution of equimolar amounts of DMPyr[SeSiMe_3_]^[20]^ and Me_3_In standing for five days at room temperature in C_6_D_6_ leads to growth of single crystals of **6**. As very slow formation of Me_4_Si is observed in the NMR spectrum, it is proposed, that **6** was formed by Se−Si bond cleavage induced via nucleophilic methyl group transfer to the Se−SiMe_3_ group in plausible anionic intermediate [Me_3_In−Se−SiMe_3_]^−^. **6** crystallizes in the monoclinic space group *P*2_1_/*n* with two ion pairs per unit cell. The three shortest interionic H‐bonds to different protons of three DMPyr^+^ cations are ranging from 2.98 Å to 2.90 Å (Figure 7).

**Figure 8:**
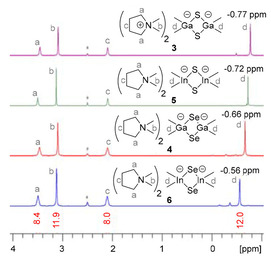
^1^H NMR spectra (**3**, 5: 500.2 MHz; 4, 5: 300.3 MHz, *dmso‐d_6_) of DMPyr_2_[Me_2_M(*μ_2_*−E)]_2_ **3**–**6**.

The ^1^H NMR spectra of the title compounds show a simple pattern of signals for two DMPyr^+^ cations and one dianion, as indicated by the corresponding integrals (Figure 8). For the dianions a general trend towards more low‐field shifted M−Me signals is observed with increasing molecular weight S<Se and Ga<In. The shifts are stronger affected by the chalcogen atoms than by the metal atoms: The M−Me protons of the selenium compounds **4** and **6** are more low‐field shifted than the corresponding signals of the sulfur homologues **3** and **5**, while the corresponding signals of the gallates **3** and **4** are slightly more high‐field shifted compared to the indates **5** and **6**.

**Figure 9:**
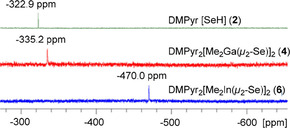
^77^Se NMR spectra (57.3 MHz, dmso‐d_6_) of the selenium containing title compounds DMPyr[SeH] (**2**, top row), DMPyr_2_[Me_2_Ga(*μ_2_*−Se)]_2_ (**4**, middle row), and DMPyr_2_[Me_2_In(*μ_2_*−Se)]_2_ (**6**, bottom row).

In contrast, ^77^Se NMR spectra show a more pronounced high‐field shift with increasing molecular weight Ga<In: The hydrochalcogenide anion in DMPyr[SeH] (**2**) displays a pronounced low‐field shift, while the anion in DMPyr_2_[Me_2_In(*μ_2_*−Se)]_2_ (**6**) is showing the strongest high‐field shifted signal (Figure 9).

### 2.3 Reactivity Studies with Chalcogenido Indates 5 and 6

#### 2.3.1 Ring Cleavage Reactions

Preliminary studies show, that these easily obtained dianionic building blocks are reactive towards other electrophilic and Lewis acidic element or metal species. The scope of using **3**–**6** as ligands to transfer [Me_2_M−E]_n_^n−^ anions to other metals and to form heteronuclear clusters has not systematically been studied so far. In this chapter we focus on reactions of the indium compounds **5** and **6** with silicon and indium electrophiles. The reason for choosing indium and not gallium homologues is the higher electron density and nucleophilicity expected at the bridging sulfido or selenido ligands in the anionic backbone [In−(μ−E)−In] compared to [Ga−(μ−E)−Ga]: Gallium has a higher Allred‐Rochow electronegativity 1.8 compared to aluminium (1.5) and indium (1.5).^[21]^ Therefore, the charge of the chalcogen dianions is believed to be much better stabilized in gallium than in indium compounds. This prognosis is reflected in the observable higher reactivity of **5** and **6** towards amphoteric electrophiles such as Me_3_Si−E‐SiMe_3_ and Me_3_In compared to **3** and **4**.

**Scheme 4:**
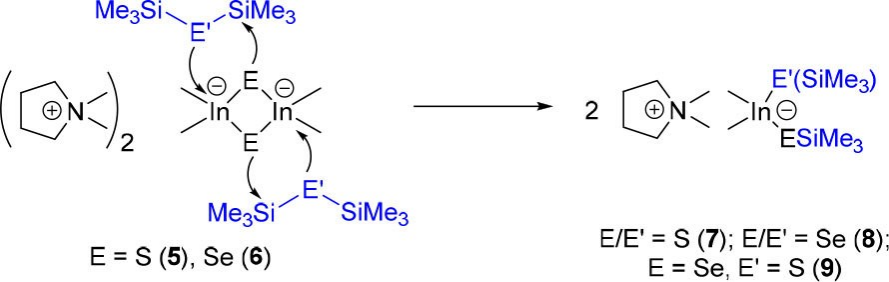
Suggested dipolar addition mechanism towards mononuclear silylchalcogenido indates 7–9.

**Figure 10:**
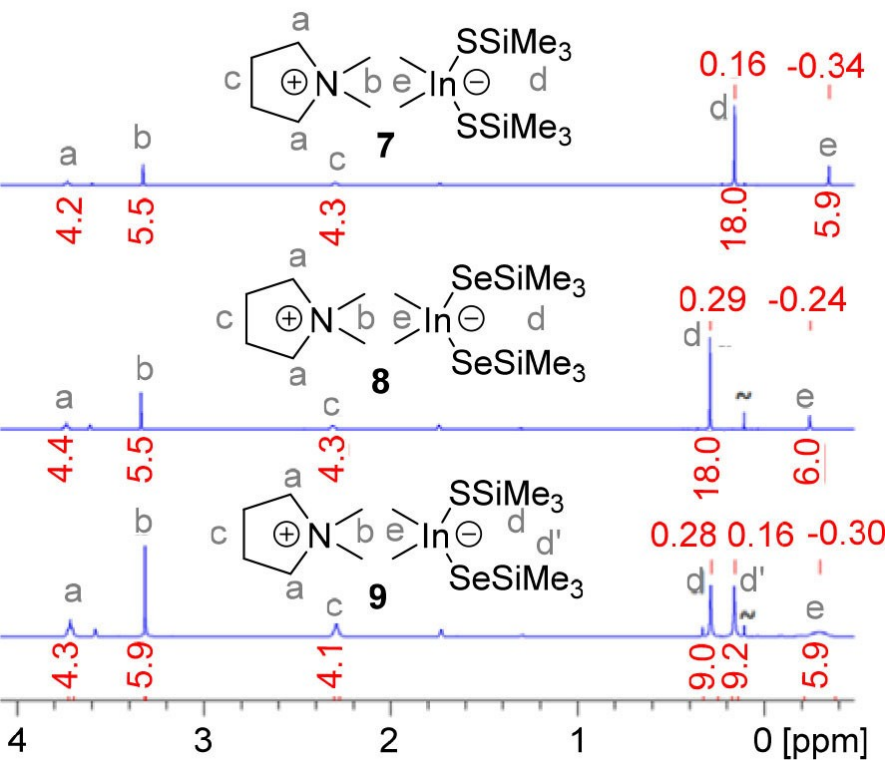
^1^H NMR spectra (250.1 MHz, *dmso‐d_6_) of the indates **7**, **8**, and **9**. The shift of the indium attached methyl groups of **9** implies the presence of a sulfido‐ and selenido‐substituted metalate anion.

The indates DMPyr_2_[Me_2_In(*μ_2_*−S)]_2_ (**5**) and DMPyr_2_[Me_2_In(*μ_2_*−Se)]_2_ (**6**) react with two equivalents of (Me_3_Si)_2_E under cleavage of the four‐membered ring to yield bis(trimethylsilylchalcogenido)‐dimethylindates DMPyr[Me_2_In(SSiMe_3_)_2_] (**7**) and DMPyr[Me_2_In(SeSiMe_3_)_2_] (**8**) (Scheme 4). A dipolar intramolecular addition mechanism is suggested, as we were able to isolate even a mixed silylsulfido‐selenido derivative DMPyr [Me_2_In(SSiMe_3_)(SeSiMe_3_)] (**9**) via selective cleavage of the ring DMPyr_2_[Me_2_In(*μ_2_*−Se)]_2_ (**6**) by addition of S(SiMe_3_)_2_. The formation of mononuclear silylchalcogenidoindates can easily be monitored by the observation of a new signal for indium attached methyl groups in the proton NMR spectra (Figure 10).

**Scheme 5:**
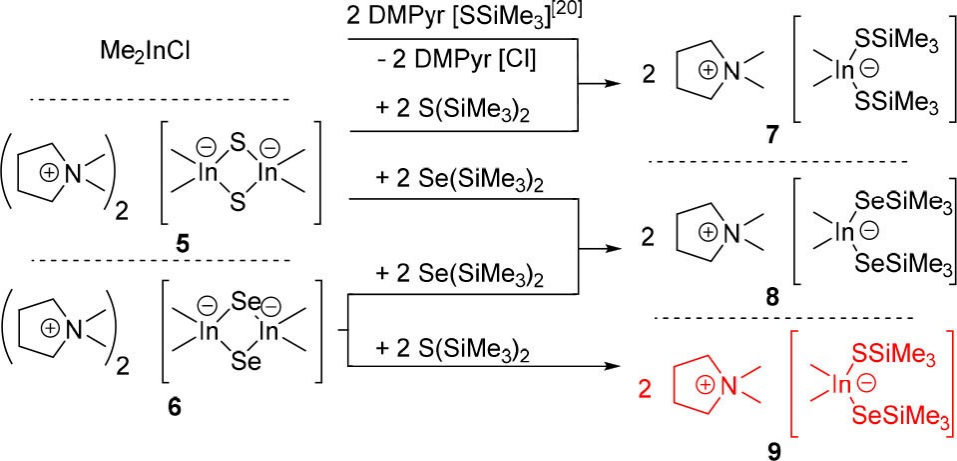
Reactions of 5 and 6 to silylchalcogenido‐dimethylindates 7, 8, and mixed 9.

Interestingly, the reaction of DMPyr_2_[Me_2_In(*μ_2_*−S)]_2_ (**5**) with >5 molar excess of Se(SiMe_3_)_2_ leads to DMPyr[Me_2_In(SeSiMe_3_)_2_] (**8**), indicating that a terminal [In−S−SiMe_3_] functionality of plausible mixed intermediate DMPyr [Me_2_In(SSiMe_3_)(SeSiMe_3_)] (**9**) can be replaced by a probably more stable [In−Se−SiMe_3_] functionality – taking also the formation of a more stable Si−S bond into consideration. Therefore, the other synthesis strategy laid out in Scheme 5 is more selective for the simple isolation of pure **9**. Finally, DMPyr[Me_2_In(SSiMe_3_)_2_] (**7**) can be prepared by reaction of two equivalents of DMPyr[SSiMe_3_] with Me_2_InCl. This strategy has the disadvantage, that another salt, DMPyr[Cl], has to be separated from much better soluble **7**, but this is the overall cheapest large scale method to synthesize such synthons for planning further condensation reaction steps with Lewis acids involving −Me and −SiMe_3_ leaving groups (Scheme 5).

Complexes containing silylchalcogenido ligands [M−E−SiMe_3_] are attractive synthons for condensation reactions e. g. with heterometal acetates M'OAc in order to selectively synthesize heteronuclear linkages M−E−M’ by elimination of Me_3_SiOAc.[^11^, ^15^, ^22^, ^23^, ^24^] For a long time, only two structural motives with heavy silylchalcogenido ligands were known: the spirocyclic compounds [(Me_2_M)_6_E(ESiMe_3_)_4_] (M=Ga, In; E=S, Se),^[25]^ and [*i*Pr_3_PCu(*μ*_*3*2_−ESiMe_3_)(InMe_3_)] (E=S, Se):^[26]^ The latter is the addition product of Me_3_In and *i*Pr_3_PCuESiMe_3_. It unites all atoms necessary to act as single‐source precursor for CuIn(S_x_Se_1−x_)_2_ materials.[^23^, ^26^] Recently, we established a series of homoleptic trielates Cat[M(ESSiMe_3_)_4_] which can act as versatile precursors for binary (Cat^+^=organic cation) and ternary materials (Cat^+^=[Cu]^+^).^[7]^

#### 2.3.2 Condensation to Higher Nuclear Clusters

**Figure 11:**
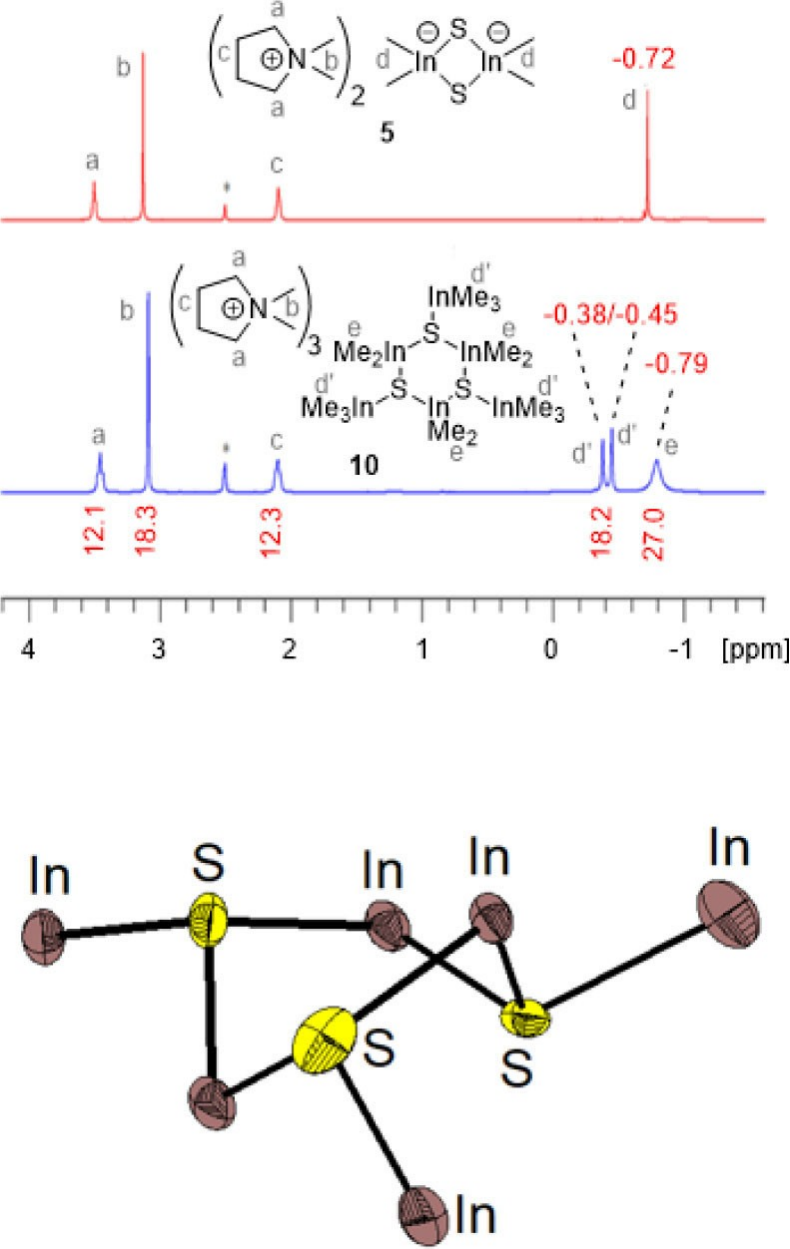
^1^H NMR spectra (500.1 MHz for **5**, 300.1 MHz for **10**, *dmso‐d_6_) of DMPyr_2_[Me_2_In(*μ_2_*‐S)]_2_ (**5**) before (top row), and after addition of two equivalents of Me_3_In. Presentation of the inner core of formed asymmetric trimer DMPyr_3_[Me_2_In(μ_2_‐S‐InMe_3_)]_3_ (**10**) with *syn‐* and *anti‐*S‐InMe_3_ groups as shown by a preliminary XRD analysis (see discussion in SI, Figure S1, page S22).

Furthermore, we investigated the reaction of Lewis base DMPyr_2_[Me_2_In(*μ_2_*−S)]_2_ (**5**) with two equivalents of trimethylindium as Lewis acid. **5** dissolved upon 1 : 2 adduct formation according to the results of an elemental analysis. Proton NMR spectra of product **10** indicated a broad signal corresponding to Me_3_In units, while Me_2_In groups are split into two signals matching in their sum with the expected 2 : 3 integral (Figure 11): This observation would be in accord with a four‐membered ring structure with either *syn‐* or *anti‐*[*μ_2_*−S−InMe_3_] bridging groups. The real situation turned out to be more interesting: Crystals of **10** were grown from THF/pentane. They were of poor quality due to multiple disorder problems, but the results of a preliminary XRD analysis allowed to determine the inner core of **10** to be a hexanuclear trimer DMPyr_3_[Me_2_In(*μ_2_*−S−InMe_3_)]_3_. The six‐membered ring adopts a twisted boat conformation with bulky terminal [*μ_2_*−S−InMe_3_] groups in *syn‐* or *anti‐*configuration with respect to each other, thus minimizing their steric interaction and releasing ring strain. This explains the proton NMR results. As a matter of fact, the highly negative charge loaded four membered ring suffered a ring expansion to a six‐membered ring upon delocalizing the high negative charge per sulfido ligand over double as much indium atoms.

**Figure 12:**
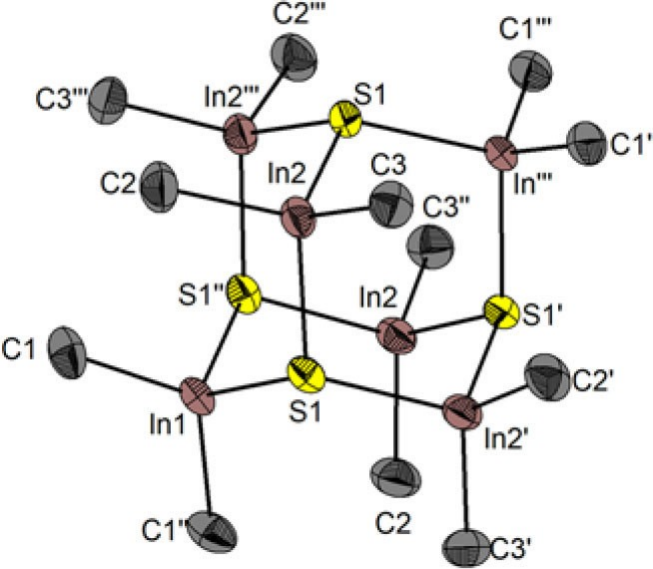
Molecular structure of the dianion in DMPyr_2_[(Me_2_In)_6_(μ_3_−S)_4_] (**11**). H‐atoms, cations, and solvent molecules are not shown for clarity. Ellipsoids shown at the 50 % level. Symmetry operations I: 3/4−y, −1/4+x, 1/4−z; II: 1−x, 1/2−y, z; III: 1/4+y, 3/4−x, 1/4−z. Selected bond lengths (in Å) and angles (in °) of the anion: S1−In1 2.522(1), S1−In2 2.522(1), S1−In2’ 2.522(1), S1’’−In1−S1 106.96(4), S1−In2−S1’’’ 107.15(4), In1−S1−In2’ 111.80(5), In1−S1−In2 109.60(5), In1−C1 2.174(5), In2−C2 2.177(6), In2−C3 2.181(5), C1−In1−C1’’ 118.5(2), C2−In2−C3 118.0(2), S1−In1−C1 108.2(1), S1−In1−C1’’ 107.3(1), In2−S1−In1−C1’’ 177.9(2), C1−In1−S1−In2’ −176.0(2), C2−In2−S1−In2’ 174.4(2), C2−In2−S1−In1 178.8(2), In1−S1−In2’−S1’ 59.65(6), In1−S1−In2‐S1’’’ −63.5(6).

**Figure 13:**
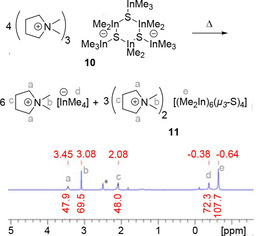
^1^H NMR spectra (300.2 MHz, *dmso‐d_6_) pointing out an assumed dismutation mechanism for the formation of **11**.

During our many attempts to grow single crystals of **10** with less disorder problems we realized, that both, the relative ratio and absolute shifts of In–CH_3_ protons changed with time after taking NMR samples. Figure 13 displays the typical proton NMR after a 14 days crystallization period in THF/pentane via a NMR sample taken in dmso‐d_6_. Repeated recrystallisation of such samples revealed, that the ratio of protons of type (d) and (e) vary a bit. This means, these signals belong to different species. However, the species with chemically equivalent In−CH_3_ protons at δ_H_=−0.64 ppm could not fully be separated from the species with δ_H_=−0.38 ppm so far: Both belong to ionic methylindate species with the same cation. Finally, a few single crystals suitable for XRD analysis were separated mechanically. They turned out to be the salt DMPyr_2_[(Me_2_In)_6_(*μ_3_*‐S)_4_] (**11**) (Figure 12). The latter crystallizes in the tetragonal space group *I*4_1_/*acd* with *Z=*8 ion pairs per unit cell and one molecule of tetrahydrofuran per formula unit. The cations show in part some disorder.

The structure refinement reveals, that a dianionic hexanuclear inverse heteroadamantane cage had been formed. In contrast to archetypical metal chalcogenide adamantane cage structures, the metals are not located in the capping positions whereas chalcogen atoms in the bridging positions, but inverse: [Me_2_In] units are bridging and sulfido ligands are capping [μ_3_−S]. Comparable inverse adamantane anion structures have been reported for the chalcogenidohydridoborates Cs[(H_2_B)_6_(*μ*_3_−E)_4_] (E=S, Se).^[27]^ A plausible path of formation of this dianion might be induced by an irreversible dissociation of Me_3_In from one of the sulfide bridges in **10**. Dissociated Me_3_In might then abstract a methyl group from a neighbouring [μ_2_−S−InMe_3_] unit forming the known tetramethylindate anion and a formally coordinatively unsaturated [In_2_S−InMe_2_]^+^ unit which is folding up with the formed nucleophilic [In−(μ−S)−In]^−^ units to build up the cluster framework. The dislocation of accumulated negative charge in such charge dissociation reactions is probably the trigger for the formation of the dianionic cluster cage. Another trigger might be the fact, that DMPyr[InMe_4_] tends to be not stable for extended periods of time. It decomposes, thus shifting the reaction from metastable **10** to more stable **11** by irreversible decay of proposed by‐product DMPyr[InMe_4_]. We tried hard to develop a synthesis method on a gram scale for **11** – without full success up to date: The thermal dissociation of metastable **10** can be accelerated in diglyme: After 18 h at 100 °C and after removing all volatiles in vacuo, the colourless solid obtained shows the same signals and similar integrals in the ^1^H NMR spectrum, which is taken as an indication for an overall stoichiometric transformation displayed in Figure 13.

**Scheme 6:**
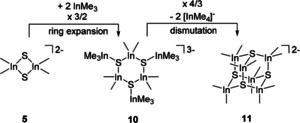
Assumed mechanism for the formation of cage compound 11 *via* 10 starting from 5.

We assume, that the formation of cluster dianion **11** with the lowest negative real charge per sulfido group is the result of accumulated charge reduction starting from low nuclearity (dinuclear) dianion **5** with the highest charge at sulfur, via metalation with Me_3_In and ring expansion towards metastable **10** and its dismutation and charge dissociation into [InMe_4_]^−^ and [(Me_2_In)_6_(μ_3_−S)_4_]^2−^ (Scheme 6).

This mechanism has to be further confirmed in future research and alternative synthesis strategies have to be developed in order to synthesize such inverse adamantane cage compounds with all combinations of Ga/In and S/Se. The SI describes first encouraging experiments indicating that the mononuclear silylchalcogenido‐dimethylindates **7**, **8** and **9** described above are indeed useful building blocks to approach clusters of type **11** under thermodynamic reaction control via condensation of metastable intermediates formed by reactions of **7**–**9** with Me_2_InCl and Me_3_In (Scheme S2 and Figures S3–S6).

## 3 Conclusions

Deprotonation of hydrochalcogenide anions of organic salts DMPyr[EH] (E=S (**1**), Se (**2**)) with trimethylgallium and indium Me_3_M (M=Ga, In) leads to a comprehensive series of dinuclear chalcogenidogallates DMPyr_2_[Me_2_Ga(*μ_2_*−E)]_2_, E=S (**3**); E=Se (**4**), and chalcogenido indates DMPyr_2_[Me_2_In(*μ_2_*−E)]_2_, E=S (**5**), Se (**6**). These are valuable building blocks for reactivity studies: Dinuclear and dianionic chalcogenido indates **5** and **6** were shown to be ring cleaved into mononuclear indates DMPyr[Me_2_In(ESiMe_3_)_2_], E=S (**7**), Se (**8**), while DMPyr_2_[Me_2_In(*μ_2_*−Se)]_2_ (**6**) is cleaved by S(SiMe_3_)_2_ to mixed silylsulfido‐selenido indate DMPyr [Me_2_In(SSiMe_3_)(SeSiMe_3_)] (**9**). By addition of two equivalents of trimethylindium to DMPyr_2_[Me_2_In(*μ_2_*−S)]_2_ (**5**) a terminal trimethylindate unit is added to each highly charged sulfur atom resulting in better charge delocalization and ring expansion into hexanuclear trianionic ring compound DMPyr_3_[Me_2_In(*μ*_2_−S−InMe_3_)]_3_ (**10**). Long term storage of **10** in solution leads to dimutation into DMPyr_2_[(Me_2_In)_6_(μ_3_−S)_4_] (**11**) displaying an interesting inverse heteroadamantane cage identified crystallographically. Based on model reactions it is likely, that **10** decomposes to **11** by thermolysis and under elimination of DMPyr[InMe_4_]. All in all, new valuable triel chalcogenido metalate building blocks and a first set of their further transformations were presented.

## Experimental Section

For methods and devices please refer to the General Consideration section in the Supporting Information.

### XRD Analyses

Please refer to the Crystallographic Information section in the Supporting Information concerning the used hardware and software used for data collection. For cell refinement and data reduction as well as structure refinement of molecular structures of chapter 3 please check Table S1–Table S5 in the Supporting Information. Deposition Number(s)1910795 (for **3**), 1910796 (for **5**), 1910797 (for **2**), 1910798 (for **1**), 1910799 (for **11**), 1910800 (for **4**), 1910794 (for **6**) contain the supplementary crystallographic data for this paper. These data are provided free of charge by the joint Cambridge Crystallographic Data Centre and Fachinformationszentrum Karlsruhe Access Structures service www.ccdc.cam.ac.uk/structures.

### Representative Synthetic Procedures

Synthetic procedures for **1**, **3**, **4**, **6**, **8**, **9** and strategies to synthesize **11** on large scale by alternative synthetic routes are provided in the Supporting Information.

### Synthesis of *N,N*‐Dimethylpyrrolidinium Hydroselenide DMPyr[SeH] (2)

Se(SiMe_3_)_2_ (6.88 g, 30.5 mmol, 1.1 eq.) was added to a solution of *N,N*‐dimethylpyrrolidinium methylcarbonat (4.86 g, 27.8 mmol, 1.0 eq) in 30 mL methanol at 0 °C. The reaction mixture was stirred for 30 min at 0 °C and for 1 hour at room temperature. All volatiles were removed in fine vacuum and the residue was diluted in acetonitrile until a saturated solution is obtained. Storing this saturated solution at −30 °C yields greenish crystals that are collected by filtration and washed two times with 10 mL diethyl ether. DMPyr[SeH] (**2**, 3.90 g, 21.6 mmol, 78 %) was obtained as slightly greenish crystals. The yield can be enhanced by further saturation of the mother liquor and subsequent recrystallisation cycles. ^1^H NMR (300.3 MHz, dmso‐*d_6_*) δ_H_=3.51 (m, 4H, (CH_3_)_2_N(CH_2_C*H*_2_)_2_), 3.14 (s, 6H, (C*H*_3_)_2_N(CH_2_CH_2_)_2_), 2.09 (m, 4H, (CH_3_)_2_N(CH_2_C*H*_2_)_2_), −6.62 (s, 1H, *H*Se) ppm. ^13^C NMR (75.5 MHz, dmso‐*d_6_*) δ_C_=64.6 (t, ^*1*^ *J_CN_*=3.2 Hz (CH_3_)_2_N(*C*H_2_CH_2_)_2_), 50.9 (t, ^*1*^ *J_CN_*=3.9 Hz, (*C*H_3_)_2_N(CH_2_CH_2_)_2_), 21.3 (s, (CH_3_)_2_N(CH_2_ *C*H_2_)_2_) ppm. ^77^Se NMR (57.3 MHz, dmso‐*d_6_*) δ_Se_=−322.9 (s, *Se*H) ppm. Anal. calcd. for C_6_H_15_N_1_Se_1_: C, 40.0; H, 8.4; N, 7.8. Found: C, 40.0; H, 8.5; N, 8.0.

### Synthesis of *N,N*‐Dimethylpyrrolidinium Dimethylsulfidoindate DMPyr_2_[Me_2_In(*μ_2_*‐S)]_2_ (5)

To a suspension of DMPyr[SH] (0.096 g, 0.73 mmol, 2.0 eq.) in 10 mL thf a solution of Me_3_In (0.121 g, 0.76 mmol, 2.1 eq.) in 10 mL thf was slowly added at −20 °C. The reaction mixture is allowed to obtain room temperature within 18 h under continuous stirring. The mixture becomes clear after approximately 15 min, and after approximately 2 hours a colorless solid precipitates. After the 18 h a colorless cloudy suspension is obtained. All volatiles were removed in fine vacuum and the residue was washed twice with 10 mL of pentane. DMPyr_2_[Me_2_MIn(*μ_2_*‐S)]_2_ (0.170 g, 0.31 mmol, 87 %) is obtained as colorless powder. ^1^H NMR (500.2 MHz, dmso‐d_6_) δ_H_=3.50 (m, 8H, (CH_3_)_2_N(CH_2_C*H*_2_)_2_), 3.13 (s, 12H, (C*H*_3_)_2_N(CH_2_CH_2_)_2_), 2.09 (m, 8H, (CH_3_)_2_N(CH_2_C*H*_2_)_2_), −0.72 (s, 12H, In(C*H*_2_)_2_×2) ppm. ^13^C NMR (125.8 MHz, dmso‐*d_6_*) δ_C_=64.6 (t, ^*1*^ *J_CN_*=3.2 Hz, (CH_3_)_2_N(*C*H_2_CH_2_)_2_), 50.9 (t, ^*1*^ *J_CN_*=3.9 Hz, (*C*H_3_)_2_N(CH_2_CH_2_)_2_), 21.3 (s, (CH_3_)_2_N(CH_2_ *C*H_2_)_2_), −0.3 (s, In(*C*H_2_)_2_) ppm. Anal. calcd. for C_16_H_40_In_2_N_2_S_2_: C, 34.7; H, 7.3; N, 5.1; S, 11.6. Found: C, 34.5; H, 7.6; N, 5.2; S, 11.3.

### Synthesis of *N,N*‐Dimethylpyrrolidinium bis(trimethylsilylsulfio)dimethylindate DMPyr[Me_2_In(SSiMe_3_)_2_] (7)

S(SiMe_3_)_2_ (0.057 g, 0.32 mmol, 1.5 eq.) is slowly added to a suspension of DMPyr_2_[Me_2_In(*μ_2_*‐S)]_2_ (**5**) (0.060 g, 0.11 mmol, 0.5 eq.) in 10 mL thf at −78 °C. The reaction mixture is slowly allowed to obtain room temperature within 18 h and stirred, until a clear solution is obtained. After removing all volatiles in fine vacuum, the oily residues are washed with 5 mL pentane and dried in fine vacuum. DMPyr[Me_2_In(SSiMe_3_)_2_] (**7**, 0.087 g, 0.20 mmol, 88 %) is obtained as colorless and oily wax. ^1^H NMR (500.2 MHz, THF‐d_8_) δ_H_=3.71 (m, 4H, (CH_3_)_2_N(CH_2_C*H*_2_)_2_), 3.31 (s, 6H, (C*H*_3_)_2_N(CH_2_CH_2_)_2_), 2.29 (m, 4H, (CH_3_)_2_N(CH_2_C*H*_2_)_2_), 0.16 (s, 18H, (H_3_C)_2_In(SSi(C*H*_3_)_3_)_2_), −0.34 (s, 6H, (*H*_3_C)_2_In(SSi(CH_3_)_3_)_2_) ppm. ^13^C NMR (125.8 MHz, THF‐d_8_) δ_C_=66.5 (t, ^*1*^ *J_CN_*=3.2 Hz, (CH_3_)_2_N(CH_2_ *C*H_2_)_2_), 52.5 (t, ^*1*^ *J_CN_*=4.0 Hz, (*C*H_3_)_2_N(CH_2_CH_2_)_2_), 22.6 (s, (CH_3_)_2_N(CH_2_ *C*H_2_)_2_), 6.5 (s, (H_3_C)_2_In(SSi(*C*H_3_)_3_)_2_), −1.9 (s, (H_3_ *C*)_2_In(SSi(CH_3_)_3_)_2_) ppm. ^29^Si NMR (99.4 MHz, THF‐d_8_) δ_Si_=8.6 (s, (H_3_C)_2_In(S*Si*(CH_3_)_3_)_2_) ppm. Anal. calcd. for C_14_H_38_InNS_2_Si_2_: C, 36.9; H, 8.4; N, 3.1; S, 14.1. Found: C, 36.9; H, 8.2; N, 3.6, S, 13.1. Note that crude product was investigated.

### Synthesis of DMPyr_3_[Me_2_In(*μ*_2_−S−InMe_3_)]_3_ (10)

To a suspension of DMPyr_2_[Me_2_In(*μ_2_*−S)]_2_ (**5**) (0.100 g, 0.180 mmol, 1.5 eq.) in 3 mL thf a solution of 0.058 g Me_3_In (0.058 g, 0.361 mmol, 3.0 eq.) in 5 mL thf was added dropwise at −78 °C. The reaction mixture was allowed to obtain room temperature within 18 h. A clear solution is obtained, that is separated from all volatiles in fine vacuum. The colorless residue is washed with 5 mL pentane and dried in fine vacuum. DMPyr_3_[Me_2_In(μ_2_−S−InMe_3_]_3_ (**10**) was obtained as colorless solid with a yield of 0.145 g (0.111 mmol, 92 %). ^1^H NMR (300.1 MHz, dmso‐d_6_) δ_H_=3.45 (m, 12H, (CH_3_)_2_N(CH_2_C*H*_2_)_2_), 3.08 (s, 18H, (C*H*_3_)_2_N(CH_2_CH_2_)_2_), 2.10 (m, 12H, (CH_3_)_2_N(CH_2_C*H*_2_)_2_), −0.38 & −0.45 (2×s, 18H, (*H*_3_C)_2_In), −0.79 (bs, 27H, μ_2_−S−In(C*H*_3_)_3_) ppm. ^13^C NMR (75.5 MHz, dmso‐d_6_) δ_C_=64.8 (t, ^*1*^ *J_CN_*=3.1 Hz, (CH_3_)_2_N(CH_2_ *C*H_2_)_2_), 51.0 (t, ^*1*^ *J_CN_*=4.1 Hz, (*C*H_3_)_2_N(CH_2_CH_2_)_2_), 21.3 (m, 12H, (CH_3_)_2_N(CH_2_ *C*H_2_)_2_), −0.79 & −0.88 (2×s, (H_3_ *C*)_2_In), −0.79 (bs, 27H, μ_2_−S−In(*C*H_3_)_3_) ppm.* Anal. calcd. for C_33_H_87_In_6_N_3_S_3_: C, 30.2; H, 6.7; N, 3.2; S, 7.3. Found: C, 30.9; H, 6.7; N, 3.5, S, 6.6. Note that crude product was investigated. *The signals in the ^13^C NMR spectrum are quite weak. The split signal for the indium attached methyl groups is hardly determinable. The ^13^C NMR signal for the μ_2_−S−InMe_3 g_roups cannot be identified clearly. This is due to a dynamic conformational rearrangement also displayed in the proton spectra.

### Synthesis of DMPyr_2_[(Me_2_In)_6_(μ_3_−S)_4_] (11)

A 30 mg sample of **10** was dissolved in THF and layered with pentane. After 14 days in the dark under argon protective gas, a crop of colorless crystals next to colorless microcrystalline material had formed. The NMR sample of a representative sample of this precipitate dissolved in dmso‐d_6_ is presented in Figure 13. It reveals a mix of what is presumed to be DMPyr[InMe_4_] and **11**. Single crystals of **11** were mechanically separated and submitted to XRD analysis. Estimated yield of separated **11**: <10 %. ^**1**^ **H NMR (300**.1 MHz, dmso‐d_6_) δ_H_=3.45 (m, 12H, (CH_3_)_2_N(CH_2_C*H*_2_)_2_), 3.08 (s, 18H, (C*H*_3_)_2_N(CH_2_CH_2_)_2_), 2.10 (m, 12H, (CH_3_)_2_N(CH_2_C*H*_2_)_2_), −0.45 (2×s, 18H, (*H*_3_C)_2_In), −0.64 (12H, μ_2_‐In(C*H*_3_)_3_) ppm.

## Conflict of interest

The authors declare no conflict of interest.

## Supporting information

As a service to our authors and readers, this journal provides supporting information supplied by the authors. Such materials are peer reviewed and may be re‐organized for online delivery, but are not copy‐edited or typeset. Technical support issues arising from supporting information (other than missing files) should be addressed to the authors.

Supplementary
