# Supplementary material for: Chalcogenido‐Dimethylgallates and ‐Indates DMPyr2[Me2M(μ2−E)]2 (M=Ga, In; E=S, Se): Building Blocks for Higher and Lower Order Chalcogenidoindates
Source: ChemistryOpen. 2021 Feb 10;10(2):83–91. doi: 10.1002/open.202000347 (PMC7874246; doi:10.1002/open.202000347)
Supplement: Supplementary file 1 — Supplementary [file OPEN-10-83-s001.pdf]

ChemistryOpen

Supporting Information

Chalcogenido-Dimethylgallates and -Indates $DMPyr_2[Me_2M(\mu_2{-}E)]_2$ (M=Ga, In; E=S, Se): Building Blocks for Higher and Lower Order Chalcogenidoindates

Jannick Guschlbauer, Tobias Vollgraff, Lars H. Finger, Klaus Harms, and Jörg Sundermeyer*

Author Contributions

J.S. Conceptualization:Lead; Funding acquisition:Lead; Validation:Equal; Writing – original draft:Supporting; Writing – review & editing:Lead
J.G. Data curation:Lead; Formal analysis:Lead; Investigation:Lead; Methodology:Equal; Visualization:Equal; Writing – original draft:Equal
L.F. Data curation:Supporting; Formal analysis:Supporting; Investigation:Supporting; Methodology:Lead
K.H. Data curation:Supporting; Formal analysis:Supporting; Validation:Supporting

Contents

Experimental Section **2**
General considerations 2
Synthesis of N,N-Dimethylpyrrolidinium hydrosulfide DMPyr[SH] (**1**) 2
Synthesis of N,N-Dimethylpyrrolidinium hydroselenide DMPyr[SeH] (**2**) 3
Synthesis of the N,N-Dimethylpyrrolidinium dimethylchalcogenidotrielates $DMPyr_2[Me_2M(\mu_2\text{-}E)]_2$ (**3-6**) 3
Synthesis of the N,N-Dimethylpyrrolidinium bis(trimethylsilylchalcogenolato)dimethylchalcogenidoindates $DMPyr[Me_2In(ESiMe_3)_2]$ (**7-9**) ... 5
NMR Spectra **8**
DMPyr[SH] (**1**) 8
DMPyr[SeH] (**2**) 9
$DMPyr_2[Me_2Ga(\mu_2\text{-}S)]_2$ (**3**) 10
$DMPyr_2[Me_2Ga(\mu_2\text{-}Se)]_2$ (**4**) 11
$DMPyr_2[Me_2In(\mu_2\text{-}S)]_2$ (**5**) 12
$DMPyr_2[Me_2In(\mu_2\text{-}Se)]_2$ (**6**) 13
$DMPyr[Me_2In(SSiMe_3)_2]$ (**7**) 14
$DMPyr[Me_2In(SeSiMe_3)_2]$ (**8**) 15
$DMPyr[Me_2In(SeSiMe_3)(SSiMe_3)]$ (**9**) 16
$DMPyr_3[Me_2In(\mu_2\text{-}S\text{-}InMe_3]_3$ (**10**) 17
Crystallographic Information **18**
XRD data for the hydrochalcogenides DMPyr[SH] (**1**) and DMPyr[SeH] (**2**) 18
XRD data for the gallates $DMPyr_2[Me_2Ga(\mu_2\text{-}S)]_2$ (**3**) and $DMPyr_2[Me_2Ga(\mu_2\text{-}Se)]_2$ (**4**) 19
XRD data for the indates $DMPyr_2[Me_2In(\mu_2\text{-}S)]_2$ (**5**) and $DMPyr_2[Me_2In(\mu_2\text{-}Se)]_2$ (**6**) 20
XRD data for the inverse heteroadamantane cage compound $DMPyr_2[(Me_2In)_6(\mu_3\text{-}S)_4]$ (**11**) 21
Structural XRD consideration for $DMPyr_3[Me_2In(\mu_2\text{-}S\text{-}InMe_3)]_3$ (**10**) 22
Alternative attempts to synthesize $DMPyr_2[(Me_2In)_6(\mu_3\text{-}S)_4]$ (11) on gram scale **24**
Thermolysis of **10** 24
A: Attempt to prepare **11** starting from **7** by elimination of $SiMe_4$ 25
B: Attempt to prepare **11** starting from **7** by elimination of $ClSiMe_3$ 26
C: Attempt to prepare **11-Ph₄P** starting from **7** by elimination of $ClSiMe_3$ 27
References **29**

Experimental section

General considerations

All preparative operations were conducted by using standard Schlenk techniques and freshly dried solvents. All solvents were dried according to common procedures[1] and passed through columns of aluminium oxide, R3-11G-catalyst (BASF) or stored over molecular sieves (3 Å or 4 Å). Other reagents were used as received unless stated otherwise. Literature known procedures were used as reported or slightly modified to synthesize DMPyr [$SSiMe_3$],[2] Me_2InCl[3] and $E(SiMe_3)_2$ (E = S, Se).[4]

Elemental analyses (C, H, N, S) were carried out by the service department for routine analysis with a vario MICRO cube (Elementar). Samples for the elemental analysis were weighted into tin capsules inside a nitrogen filled glovebox. ^1H and proton decoupled ^{13}C-NMR spectra were recorded in automation with a Bruker Avance II 300 spectrometer, ^{29}Si- and ^{77}Se-NMR spectra were recorded by the service department for NMR analyses with a Bruker Avance II HD 300, DRX 400 or Avance III 500 spectrometer. All spectra were recorded at ambient temperature. ^1H- and ^{13}C-NMR spectra were calibrated using residual proton signals of the solvent (dmso-d_6: δ_H 2.50 ppm, δ_C 39.52 ppm, thf-d_8: δ_H 3.58 & 1.72 ppm, δ_C 67.21 & 25.31 ppm). ^{29}Si-NMR spectra were referenced externally ($SiMe_4$: δ_{Si} 0.00 ppm) just as ^{77}Se-NMR spectra (Me_2Se δ_{Se} 0.00 ppm).

*Synthesis of N,N-Dimethylpyrrolidinium hydrosulfide DMPyr[SH] (**1**)*

A constant flow of hydrogen sulfide was fed into a solution of *N,N*-dimethylpyrrolidinium methylcarbonat (7.00 g, 40.0 mmol, 1.0 eq.) in 30 mL methanol for 30 minutes at room temperature. After flushing the solution with a constant flow of argon for 5 minutes all volatiles were removed in fine vacuum. The residue was recrystallized from a acetonitrile/diethyl ether mixture at –30 °C. After filtration and washing the residue for two times with 10 mL diethyl ether the crystals were dried in fine vacuum. DMPyr[SH] (**1**, 3.68 g, 27.6 mmol, 69%) was obtained as colorless solid. The yield can be enhanced by further saturation of the mother liquor and subsequent crystalisation cycles.

^{1}H-NMR (300.1 MHz, dmso-d_6) δ_H = 3.51 (m, 4H, $(CH_3)_2N(CH_2CH_2)_2$), 3.14 (s, 6H, $(CH_3)_2N(CH_2CH_2)_2$), 2.08 (m, 4H, $(CH_3)_2N(CH_2CH_2)_2$), –4.04 (s, 1H, *H*S) ppm. **^{13}C-NMR** (75.5 MHz, dmso-d_6) δ_C = 64.7(t, $^1J_{CN}$ = 3.0 Hz $(CH_3)_2N(CH_2CH_2)_2$), 50.8 (t, $^1J_{CN}$ = 3.8 Hz, $(CH_3)_2N(CH_2CH_2)_2$), 21.3 (s, $(CH_3)_2N(CH_2CH_2)_2$) ppm. **Anal. calcd.** for $C_6H_{15}N_1S_1$: C, 54.1; H, 11.4; N, 10.5; S, 24.1. Found: C, 53.9; H, 11.4; N, 10.6; S, 25.2.

*Synthesis of N,N-Dimethylpyrrolidinium hydroselenide DMPyr[SeH] (**2**)*

$Se(SiMe_3)_2$ (6.88 g, 30.5 mmol, 1.1 eq.) was added to a solution of *N,N*-dimethylpyrrolidinium methylcarbonat (4.86 g, 27.8 mmol, 1.0 eq) in 30 mL methanol at 0 °C. The reaction mixture was stirred for 30 min at 0 °C and for 1 hour at room temperature. All volatiles were removed in fine vacuum and the residue was diluted in acetonitrile until a saturated solution is obtained. Storing this saturated solution at –30 °C yields greenish crystals that are collected by filtration and washed two times with 10 mL diethyl ether. DMPyr[SeH] (**2**, 3.90 g, 21.6 mmol, 78%) was obtained as slightly greenish crystals. The yield can be enhanced by further saturation of the mother liquor and subsequent recrysalisation cycles.

^{1}H-NMR (300.3 MHz, dmso-*d*$_6$) δ_H = 3.51 (m, 4H, $(CH_3)_2N(CH_2C\mathit{H}_2)_2$), 3.14 (s, 6H, $(C\mathit{H}_3)_2N(CH_2CH_2)_2$), 2.09 (m, 4H, $(CH_3)_2N(CH_2C\mathit{H}_2)_2$), –6.62 (s, 1H, *H*Se) ppm. **^{13}C-NMR** (75.5 MHz, dmso-*d*$_6$) δ_C = 64.6 (t, $^{1}J_{CN}$ = 3.2 Hz $(CH_3)_2N(\mathit{C}H_2CH_2)_2$), 50.9 (t, $^{1}J_{CN}$ = 3.9 Hz, $(\mathit{C}H_3)_2N(CH_2CH_2)_2$), 21.3 (s, $(CH_3)_2N(CH_2\mathit{C}H_2)_2$) ppm. **^{77}Se-NMR** (57.3 MHz, dmso-*d*$_6$) δ_{Se} = –322.9 (s, *Se*H) ppm. **Anal. calcd.** for $C_6H_{15}N_1Se_1$: C, 40.0; H, 8.4; N, 7.8. Found: C, 40.0; H, 8.5; N, 8.0.

*Synthesis of the N,N-Dimethylpyrrolidinium dimethylchalcogenidotrielates $DMPyr_2[Me_2M(\mu_2\text{-}E)]_2$ (**3-6**)*

As the title compounds **3-6** can be prepared analogly, the general procedure is described.

To a suspension of **DMPyr[EH]** in 10 mL thf a solution of **Me_3M** in 10 mL thf was slowly added at –20 °C. The reaction mixture is allowed to obtain room temperature within 18 h under continuous stirring. The mixture becomes clear after approximately 15 min, and after approximately 2 hours a colorless solid precipitates. After the 18 h a colorless cloudy suspension is obtained. All volatiles were removed in fine vacuum and the residue was washed twice with 10 mL of pentane. The target compound **$DMPyr_2[Me_2M(\mu_2\text{-}E)]_2$** is obtained as colorless powder.

Used amounts of educts and yields of the syntheses of 3-6:

$DMPyr_2[Me_2M(\mu_2\text{-}E)]_2$	DMPyr[EH]	Me_3M	Yield
**$DMPyr_2[Me_2Ga(\mu_2\text{-}S)]_2$ (3)**	E = S<br>0.115 g, 0.86 mmol, 2.0 eq.	M = Ga<br>0.104 g, 0.90 mmol, 2.1 eq.	0.193 g,<br>0.41 mmol, 96%
**$DMPyr_2[Me_2Ga(\mu_2\text{-}Se)]_2$ (4)**	E = Se<br>0.129 g, 0.72 mmol, 2.0 eq.	M = Ga<br>0.129 g, 0.72 mmol, 2.1 eq.	0.191 g<br>0.34 mmol, 94%
**$DMPyr_2[Me_2In(\mu_2\text{-}S)]_2$ (5)**	E = S<br>0.096 g, 0.72 mmol, 2.0 eq.	M = In<br>0.121 g, 0.76 mmol, 2.1 eq.	0.170 g,<br>0.31 mmol, 87%
**$DMPyr_2[Me_2In(\mu_2\text{-}Se)]_2$ (6)**	E = Se<br>0.111 g, 0.62 mmol, 2.0 eq	M = In<br>0.104 g, 0.65 mmol, 2.1 eq.	0.183 g,<br>0.28 mmol, 92%

DMPyr$_2$[Me$_2$Ga(μ_2-S)]$_2$ (3)

^{1}H-NMR (500.1 MHz, dmso-d$_6$) δ_H = 3.45 (m, 8H, (CH$_3$)$_2$N(CH$_2$C*H*$_2$)$_2$), 3.09 (s, 12H, (C*H*$_3$)$_2$N(CH$_2$CH$_2$)$_2$), 2.09 (m, 8H, (CH$_3$)$_2$N(CH$_2$C*H*$_2$)$_2$), –0.77 (s, 12H, Ga(C*H*$_2$)$_2$ x 2) ppm. **^{13}C NMR** (125.8 MHz, dmso-*d$_6$*) δ_C = 64.7 (t, $^1J_{CN}$ = 3.0 Hz, (CH$_3$)$_2$N(*C*H$_2$CH$_2$)$_2$), 50.9 (t, $^1J_{CN}$ = 3.8 Hz, (*C*H$_3$)$_2$N(CH$_2$CH$_2$)$_2$), 21.3 (s, (CH$_3$)$_2$N(CH$_2$*C*H$_2$)$_2$),), –0.4 (s, Ga(*C*H$_2$)$_2$ x 2) ppm. **Anal. calcd.** for $C_{16}H_{40}Ga_2N_2S_2$: C, 41.4; H, 8.7; N, 6.0; S, 13.8. Found: C, 42.3; H, 9.5; N, 6.3; S, 12.9. Note that the crude highly air sensitive powder and not single crystals were submitted to combustion analyses. Deviations might be traced back on the slight excess of Me$_3$Ga used.

DMPyr$_2$[Me$_2$Ga(μ_2-Se)]$_2$ (4)

^{1}H-NMR (300.2 MHz, dmso-d$_6$) δ_H = 3.46 (m, 8H, (CH$_3$)$_2$N(CH$_2$C*H*$_2$)$_2$), 3.10 (s, 12H, (C*H*$_3$)$_2$N(CH$_2$CH$_2$)$_2$), 2.10 (m, 8H, (CH$_3$)$_2$N(CH$_2$C*H*$_2$)$_2$), –0.66 (s, 12H, Ga(C*H*$_2$)$_2$ x 2) ppm. **^{13}C-NMR** (125.8 MHz, dmso-*d$_6$*) δ_C = 64.7 (t, $^1J_{CN}$ = 2.9 Hz, (CH$_3$)$_2$N(*C*H$_2$CH$_2$)$_2$), 51.0 (t, $^1J_{CN}$ = 3.8 Hz, (*C*H$_3$)$_2$N(CH$_2$CH$_2$)$_2$), 21.3 (s, (CH$_3$)$_2$N(CH$_2$*C*H$_2$)$_2$), –1.1 (s, Ga(*C*H$_2$)$_2$) ppm. **^{77}Se-NMR** (57.3 MHz, dmso-d$_6$) δ_{Se} = -335.2 (s, [Me$_2$Ga(μ_2-*Se*)]$_2^{2-}$) ppm. **Anal. calcd.** for $C_{16}H_{40}Ga_2N_2Se_2$: C, 34.5; H, 7.2; N, 5.0. Found: C, 35.9; H, 8.2; N, 5.0. Note that the crude highly air sensitive powder and not single crystals were submitted to combustion analyses. Deviations might be traced back on the slight excess of Me$_3$Ga used.

DMPyr$_2$[Me$_2$In(μ_2-S)]$_2$ (5)

^{1}H-NMR (500.2 MHz, dmso-d$_6$) δ_H = 3.50 (m, 8H, (CH$_3$)$_2$N(CH$_2$C*H*$_2$)$_2$), 3.13 (s, 12H, (C*H*$_3$)$_2$N(CH$_2$CH$_2$)$_2$), 2.09 (m, 8H, (CH$_3$)$_2$N(CH$_2$C*H*$_2$)$_2$), –0.72 (s, 12H, In(C*H*$_2$)$_2$ x 2) ppm. **^{13}C-NMR** (125.8 MHz, dmso-*d$_6$*) δ_C = 64.6 (t, $^1J_{CN}$ = 3.2 Hz, (CH$_3$)$_2$N(*C*H$_2$CH$_2$)$_2$), 50.9 (t, $^1J_{CN}$ = 3.9 Hz, (*C*H$_3$)$_2$N(CH$_2$CH$_2$)$_2$), 21.3 (s, (CH$_3$)$_2$N(CH$_2$*C*H$_2$)$_2$), –0.3 (s, In(*C*H$_2$)$_2$) ppm. **Anal. calcd.** for $C_{16}H_{40}In_2N_2S_2$: C, 34.7; H, 7.3; N, 5.1; S, 11.6. Found: C, 34.5; H, 7.6; N, 5.2; S, 11.3.

DMPyr$_2$[Me$_2$In(μ_2-Se)]$_2$ (6)

^{1}H-NMR (300.2 MHz, dmso-d$_6$) δ_H = 3.49 (m, 8H, (CH$_3$)$_2$N(CH$_2$C*H*$_2$)$_2$), 3.12 (s, 12H, (C*H*$_3$)$_2$N(CH$_2$CH$_2$)$_2$), 2.10 (m, 8H, (CH$_3$)$_2$N(CH$_2$C*H*$_2$)$_2$), –0.56 (s, 12H, In(C*H*$_2$)$_2$ x 2) ppm. **^{13}C-NMR** (125.8 MHz, dmso-*d$_6$*) δ_C = 64.7 (t, $^1J_{CN}$ = 3.0 Hz, (CH$_3$)$_2$N(*C*H$_2$CH$_2$)$_2$), 50.9 (t, $^1J_{CN}$ = 3.8 Hz, (*C*H$_3$)$_2$N(CH$_2$CH$_2$)$_2$), 21.3 (s, (CH$_3$)$_2$N(CH$_2$*C*H$_2$)$_2$), –0.0 (s, In(*C*H$_2$)$_2$) ppm. **^{77}Se-NMR** (57.3 MHz, dmso-d$_6$) δ_H = –470.0 (s, [Me$_2$Ga(μ_2-*Se*)]$_2^{2-}$) ppm. **Anal. calcd.** for $C_{16}H_{40}In_2N_2Se_2$: C, 29.7; H, 6.2; N, 4.3. Found: C, 29.7; H, 6.0; N, 4.7.

*Synthesis of the N,N-Dimethylpyrrolidinium bis(trimethylsilylchalcogenolato)dimethylchalcogenidoindates DMPyr[$Me_2In(ESiMe_3)_2$] (**7-9**)*

As complexes with $ESiMe_3$-moiety show a pronounced instability towards some common solvents, the usage was restricted on inert ethers and aliphats. As chlorinated solvents and dmso-d_6 decompose the target anions, the usage of THF-d_8 was indicated for NMR-analysis. Our first method to prepare DMPyr [$Me_2In(SSiMe_3)_2$] (**7**) was the addition/substitution reaction between one equivalent of Me_2InCl and two equivalents of DMPyr [$SSiMe_3$]. After separation of one equivalent of the byproduct DMPyr [Cl] the target molecule is obtained. By silylation of the indate dianion $DMPyr_2[Me_2In(\mu_2\text{-}S)]_2$ (**5**) with $S(SiMe_3)_2$ no byproducts emerge, and the pure target compound is obtained quantitatively by simple removing all volatiles in fine vacuum. For both methods the target compounds could be obtained in purely and quantitatively, but the latter one is way more convenient, and also allows the preparation of the mixed substituted compound DMPyr [$Me_2In(SSiMe_3)(SeSiMe_3)$] (**7**).

Preparation of DMPyr[$Me_2In(SSiMe_3)_2$] (7) by addition/substitution of DMPyr[$SSiMe_3$] to Me_2InCl

To a suspension of DMPyr [$SSiMe_3$] (0.36 g, 1.76 mmol, 2.0 eq.) in 5 mL thf a solution of Me_2InCl (0.16 g, 0.88 mmol, 1.0 eq.) is added dropwise at –78 °C. The reaction mixture is slowly warmed to room temperature within 18 h. the suspension is filtered. All volatiles were removed from the filtrate in fine vacuum, and the colorless oily residue was washed with 5 mL pentane, and the residue dried in fine vacuum. The target compound is obtained as colorless wax. The identity of the product was proven via ^{1}H-NMR and ^{29}Si-NMR that were identical to those of **7** prepared by the other method.

*Preparation of DMPyr [$Me_2In(ESiMe_3)(E'SiMe_3)$] (**7-9**) by addition of E'$(SiMe_3)_2$ to **3-6***

E'$(SiMe_3)_2$ is slowly added to a suspension of $DMPyr_2[Me_2In(\mu_2\text{-}E)]_2$ in 10 mL thf at –78 °C. The reaction mixture is slowly allowed to obtain room temperature within 18 h and stirred, until a clear solution is obtained. After removing all volatiles in fine vacuum, the oily residues are washed with 5 mL pentane and dried in fine vacuum. The target compounds **7-9** are obtained as colorless waxes.

Used amounts of educts and yields of the syntheses of 7-9:

DMPyr [$Me_2In(ESiMe_3)(E'SiMe_3)$]	$DMPyr_2[Me_2In(\mu_2\text{-}E)]_2$	E'$(SiMe_3)_2$	Yield
**E = E' = S (7)**	E = S<br>0.060 g, 0.11 mmol, 0.5 eq.	E' = S<br>0.057 g, 0.32 mmol, 1.5 eq.	0.087 g,<br>0.20 mmol, 88%
**E = E' = Se (8)**	E = Se<br>0.060 g, 0.09 mmol, 0.5 eq.	E' = Se<br>0.063 g, 0.28 mmol, 1.5 eq.	0.092 g<br>0.17 mmol, 90%
**E = Se, E' = S (9)**	E = Se<br>0.060 g, 0.09 mmol, 0.5 eq.	E' = S<br>0.050 g, 0.28 mmol, 1.5 eq.	0.081 g,<br>0.16 mmol, 87%

DMPyr[Me$_2$In(SSiMe$_3$)$_2$] (7)

^{1}H-NMR (500.2 MHz, THF-d$_8$) δ_H = 3.71 (m, 4H, (CH$_3$)$_2$N(CH$_2$C*H*$_2$)$_2$), 3.31 (s, 6H, (C*H*$_3$)$_2$N(CH$_2$CH$_2$)$_2$), 2.29 (m, 4H, (CH$_3$)$_2$N(CH$_2$C*H*$_2$)$_2$), 0.16 (s, 18H, (H$_3$C)$_2$In(SSi(C*H*$_3$)$_3$)$_2$), –0.34 (s, 6H, (*H*$_3$C)$_2$In(SSi(CH$_3$)$_3$)$_2$) ppm. **^{13}C-NMR** (125.8 MHz, THF-d$_8$) δ_C = 66.5 (t, $^1J_{CN}$ = 3.2 Hz, (CH$_3$)$_2$N(CH$_2$*C*H$_2$)$_2$), 52.5 (t, $^1J_{CN}$ = 4.0 Hz, (*C*H$_3$)$_2$N(CH$_2$CH$_2$)$_2$), 22.6 (s, (CH$_3$)$_2$N(CH$_2$*C*H$_2$)$_2$), 6.5 (s, (H$_3$C)$_2$In(SSi(*C*H$_3$)$_3$)$_2$), –1.9 (s, (H$_3$*C*)$_2$In(SSi(CH$_3$)$_3$)$_2$) ppm. **^{29}Si-NMR** (99.4 MHz, THF-d$_8$) δ_{Si} = 8.6 (s, (H$_3$C)$_2$In(S*Si*(CH$_3$)$_3$)$_2$) ppm. **Anal. calcd.** for $C_{14}H_{38}InNS_2Si_2$: C, 36.9; H, 8.4; N, 3.1; S, 14.1. Found: C, 36.9; H, 8.2; N, 3.6, S, 13.1.

DMPyr[Me$_2$In(SeSiMe$_3$)$_2$] (8)

^{1}H-NMR (500.1 MHz, THF-d$_8$) δ_H = 3.71 (m, 4H, (CH$_3$)$_2$N(CH$_2$C*H*$_2$)$_2$), 3.31 (s, 6H, (C*H*$_3$)$_2$N(CH$_2$CH$_2$)$_2$), 2.29 (m, 4H, (CH$_3$)$_2$N(CH$_2$C*H*$_2$)$_2$), 0.29 (s, 18H, (H$_3$C)$_2$In(SeSi(C*H*$_3$)$_3$)$_2$), –0.24 (s, 6H, (*H*$_3$C)$_2$In(SeSi(CH$_3$)$_3$)$_2$) ppm. **^{13}C-NMR** (125.8 MHz, THF-d$_8$) δ_C = 66.7 (t, $^1J_{CN}$ = 3.1 Hz, (CH$_3$)$_2$N(CH$_2$*C*H$_2$)$_2$), 52.7 (t, $^1J_{CN}$ = 4.0 Hz, (*C*H$_3$)$_2$N(CH$_2$CH$_2$)$_2$), 22.6 (s, (CH$_3$)$_2$N(CH$_2$*C*H$_2$)$_2$), 6.9 (s, (H$_3$C)$_2$In(SeSi(*C*H$_3$)$_3$)$_2$ ppm.* **Anal. calcd.** for $C_{14}H_{38}InNSe_2Si_2$: C, 30.6; H, 7.0; N, 2.6. Found: C, 30.3; H, 6.6; N, 3.0.

*The signal for the indium attached methyl groups could not be identified in the ^{13}C-NMR spectrum, as the concentration of the saturated solution of **8** in THF-d$_8$ is not high enough. No ^{29}Si- and ^{77}Se-NMR spectra were obtained for this reason.

DMPyr[Me$_2$In(SeSiMe$_3$)(SSiMe$_3$)$_2$] (9)

^{1}H-NMR (500.1 MHz, THF-d$_8$) δ_H = 3.71 (m, 4H, (CH$_3$)$_2$N(CH$_2$C*H*$_2$)$_2$), 3.31 (s, 6H, (C*H*$_3$)$_2$N(CH$_2$CH$_2$)$_2$), 2.29 (m, 4H, (CH$_3$)$_2$N(CH$_2$C*H*$_2$)$_2$), 0.28 (s, 9H, (H$_3$C)$_2$In(SeSi(C*H*$_3$)$_3$)(SSi(CH$_3$)$_3$)), 0.16 (s, 9H, (H$_3$C)$_2$In(SeSi(CH$_3$)$_3$)(SSi(C*H*$_3$)$_3$)), –0.30 (s, 6H, (*H*$_3$C)$_2$In(SeSi(CH$_3$)$_3$)(SSi(C*H*$_3$)$_3$))) ppm. **^{13}C-NMR** (125.8 MHz, THF-d$_8$) δ_C = 66.6 (t, $^1J_{CN}$ = 3.1 Hz, (CH$_3$)$_2$N(CH$_2$*C*H$_2$)$_2$), 52.6 (t, $^1J_{CN}$ = 4.0 Hz, (*C*H$_3$)$_2$N(CH$_2$CH$_2$)$_2$), 22.6 (s, (CH$_3$)$_2$N(CH$_2$*C*H$_2$)$_2$), 6.9 (s, (H$_3$C)$_2$In(SeSi(*C*H$_3$)$_3$)(SSi(CH$_3$)$_3$)), 6.5 (s, (H$_3$C)$_2$In(SeSi(CH$_3$)$_3$)(SSi(*C*H$_3$)$_3$)) ppm.* **Anal. calcd.** for $C_{14}H_{38}InNSeSSi_2$: C, 33.5; H, 7.6; N, 2.6, S, 6.4. Found: C, 32.4; H, 6.8; N, 3.4; S, 4.9. Note that the crude product was investigated. The oily appearance of the product made washing processes and the exact preparation of the elemental analysis sample difficult. Deviations might be traced back on small amounts of unreacted (DMPyr$_2$[Me$_2$In(μ_2-Se)]$_2$ (**6**)).

*The signal for the indium attached methyl groups could hardly be identified, due to the low concentration of the saturated solution of **9** in THF-d$_8$. Therefore also no ^{29}Si- and no ^{77}Se-NMR spectra could be obtained.

*Preparation of $DMPyr_3[Me_2In(\mu_2\text{-}S\text{-}InMe_3)]_3$ (**10**)*

To a suspension of $DMPyr_2[Me_2In(\mu_2\text{-}S)]_2$ (**5**) (0.100 g, 0.180 mmol, 1.5 eq.) in 3 mL thf a solution of 0.058 g Me_3In (0.058 g, 0.361 mmol, 3.0 eq.) in 5 mL thf was added dropwise at –78 °C. The reaction mixture was allowed to obtain room temperature within 18 h. A clear solution is obtained, that is separated from all volatiles in fine vacuum. The colorless residue is washed with 5 mL pentane and dried in fine vacuum. $DMPyr_3[Me_2In(\mu_2\text{-}S\text{-}InMe_3)]_3$ (**10**) was obtained as colorless solid with a yield of 0.145 g (0.111 mmol, 92%).

^{1}H-NMR (300.1 MHz, dmso-d$_6$) δ_H = 3.45 (m, 12H, $(CH_3)_2N(CH_2CH_2)_2$), 3.08 (s, 18H, $(CH_3)_2N(CH_2CH_2)_2$), 2.10 (m, 12H, $(CH_3)_2N(CH_2CH_2)_2$), –0.38 & –0.45 (2 x s, 18H, $(H_3C)_2In$), –0.79 (bs, 27H, μ_3-S-In$(CH_3)_3$) ppm. **^{13}C-NMR** (75.5 MHz, dmso-d$_6$) δ_C = 64.8 (t, $^1J_{CN}$ = 3.1 Hz, $(CH_3)_2N(CH_2CH_2)_2$), 51.0 (t, $^1J_{CN}$ = 4.1 Hz, $(CH_3)_2N(CH_2CH_2)_2$), 21.3 (m, 12H, $(CH_3)_2N(CH_2CH_2)_2$), –0.79 & –0.88 (2 x s, $(H_3C)_2In$), –0.79 (bs, 27H, μ_3-S-In$(CH_3)_3$) ppm.* **Anal. calcd.** for $C_{33}H_{87}In_6N_3S_3$: C, 30.2; H, 6.7; N, 3.2; S, 7.3. Found: C, 30.9; H, 6.7; N, 3.5, S, 6.6. Note that crude product was investigated.

*The signals in the ^{13}C-NMR spectrum are quite weak. The multiplicity of the N-attached carbon atoms was determined manually. The split signal for the indium attached methyl groups is hardly determinable. The ^{13}C-NMR-signal for the μ_2-SInMe$_3$ groups cannot be identified clearly due to dynamic conformational changes in solution.

NMR spectra

*DMPyr[SH] (**1**)*

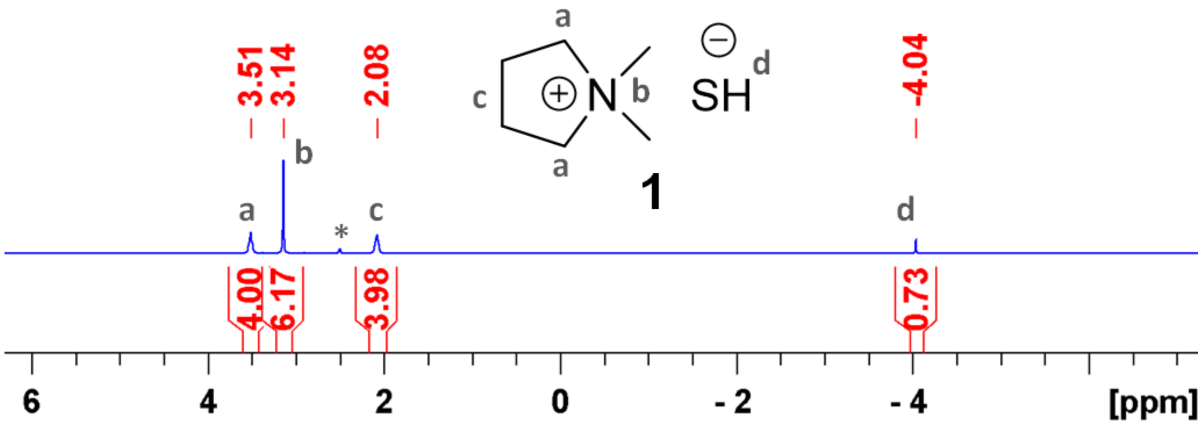

^{1}**H-NMR** (300.3 MHz, *dmso-d_6) of DMPyr [SH] (**1**)

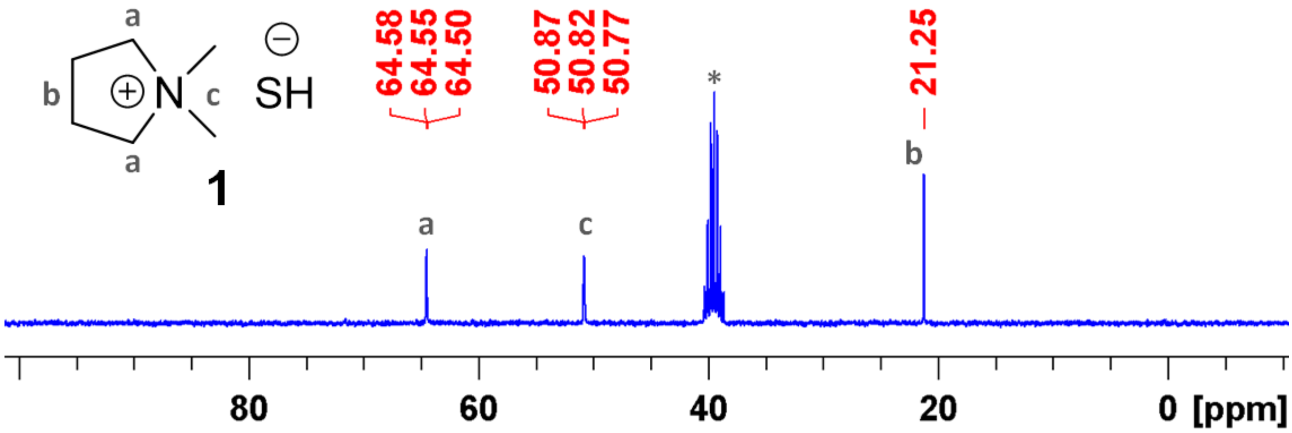

^{13}**C-NMR** (75.5 MHz, *dmso-d_6) of DMPyr [SH] (**1**)

*DMPyr[SeH] (**2**)*

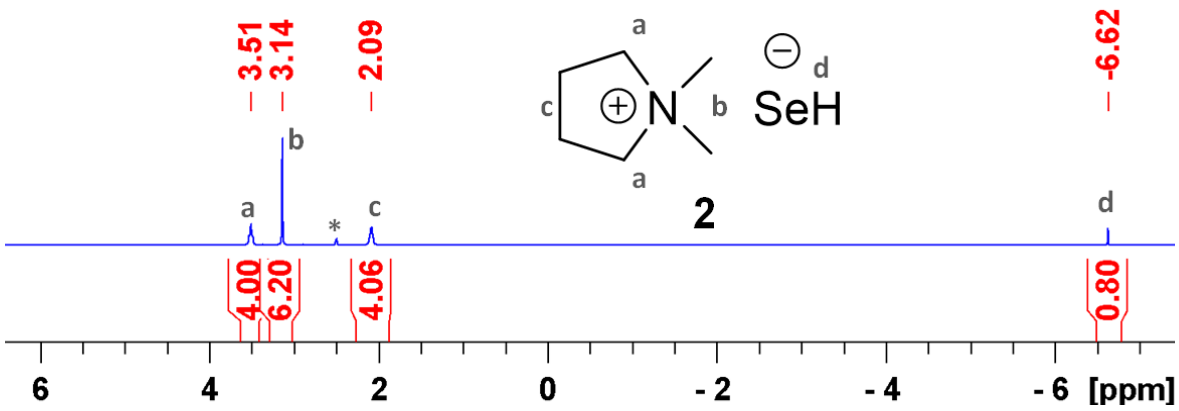

^{1}H-NMR (300.3 MHz, *dmso-d_6) of DMPyr [SeH] (**2**).

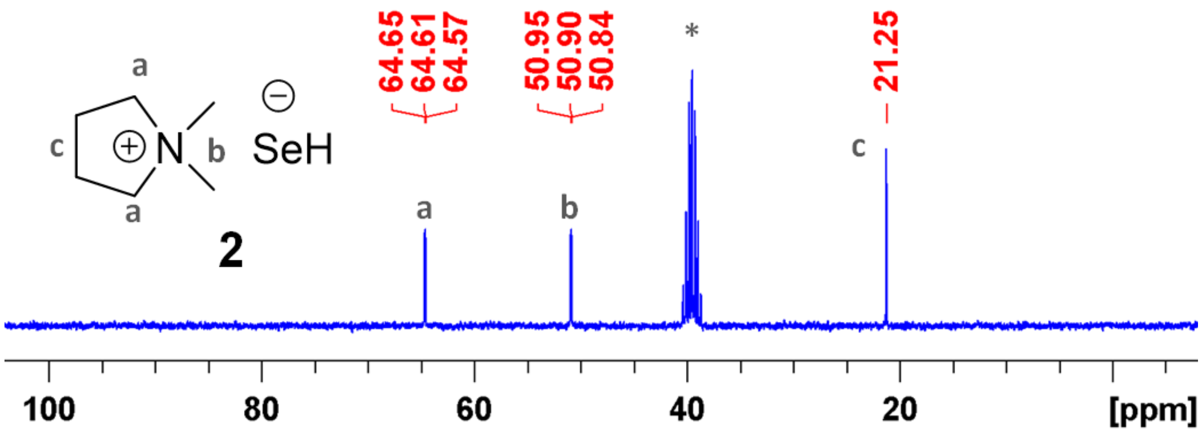

^{13}C-NMR (172.1 MHz, *dmso-d_6) of DMPyr [SeH] (**2**).

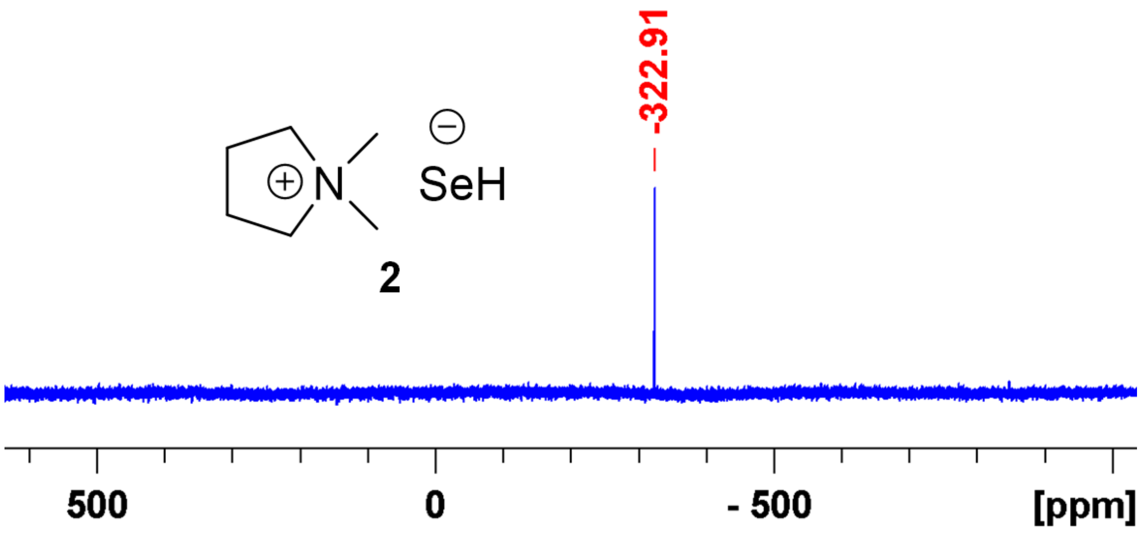

^{77}Se-NMR (57.3 MHz, dmso-d_6) of DMPyr [SeH] (**2**).

DMPyr

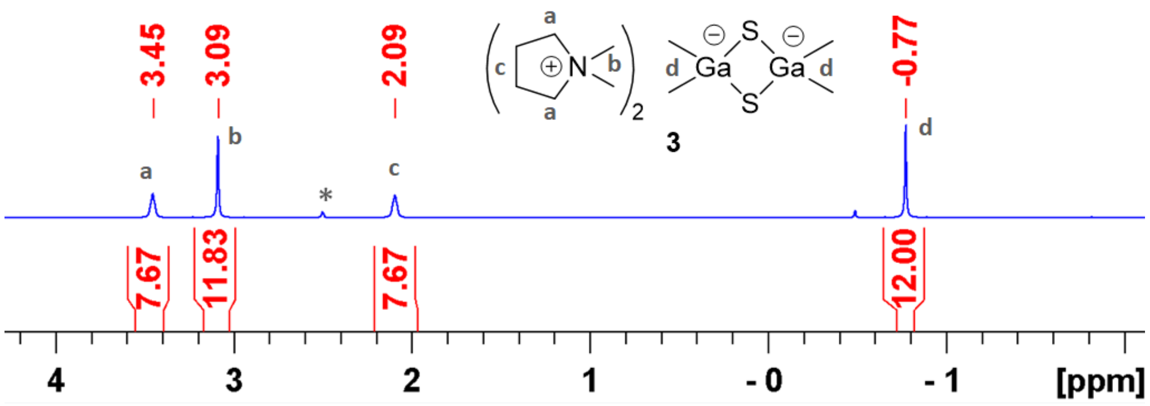

^{1}H-NMR (500.1 MHz, *dmso-d$_6$) of DMPyr$_2$[Me$_2$Ga(μ_2-S)]$_2$ (**3**).

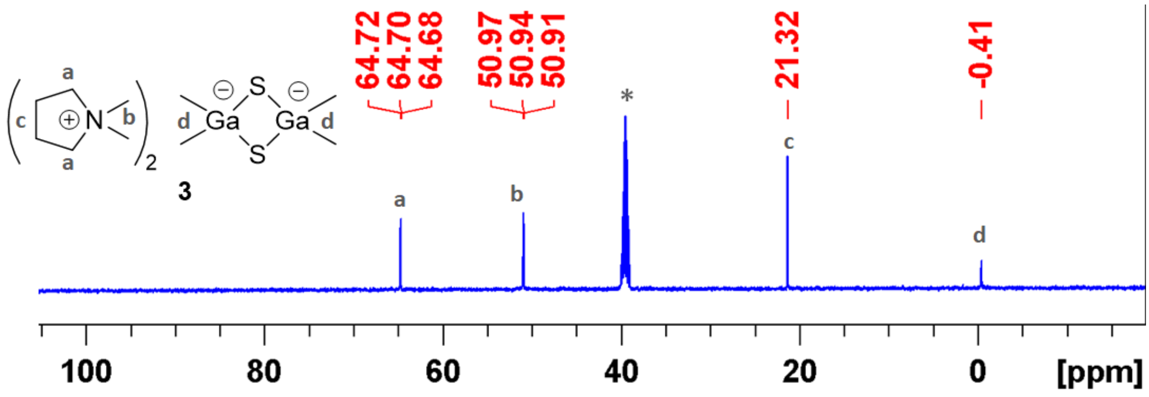

^{13}C-NMR (125.8 MHz, *dmso-*d$_6$*) of DMPyr$_2$[Me$_2$Ga(μ_2-S)]$_2$ (**3**).

DMPyr$_2$[Me$_2$Ga(μ_2-Se)]$_2$ (4)

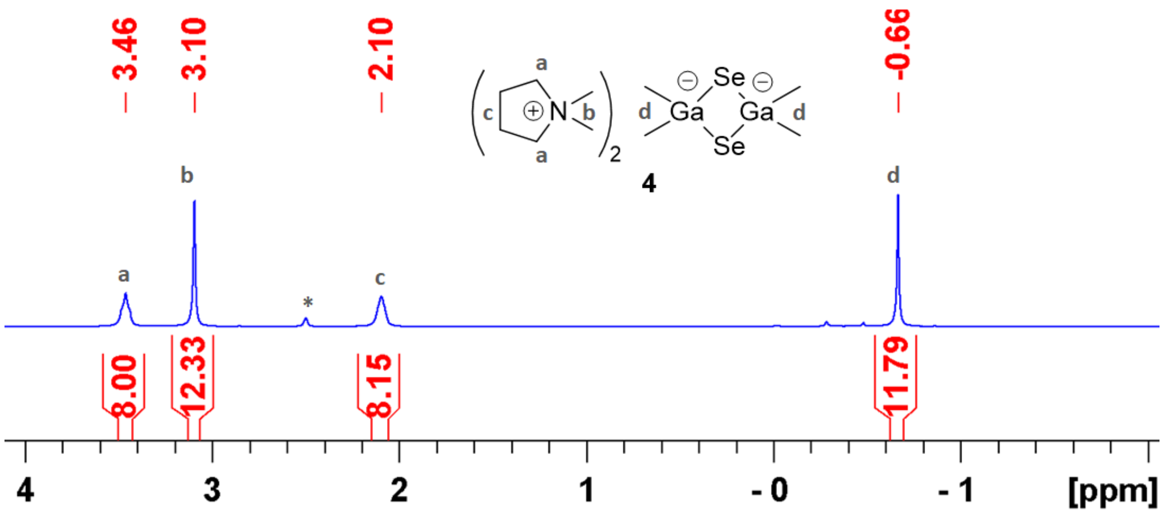

^{1}H-NMR (300.1MHz, *dmso-d$_6$) DMPyr$_2$[Me$_2$Ga(μ_2-Se)]$_2$ (**4**).

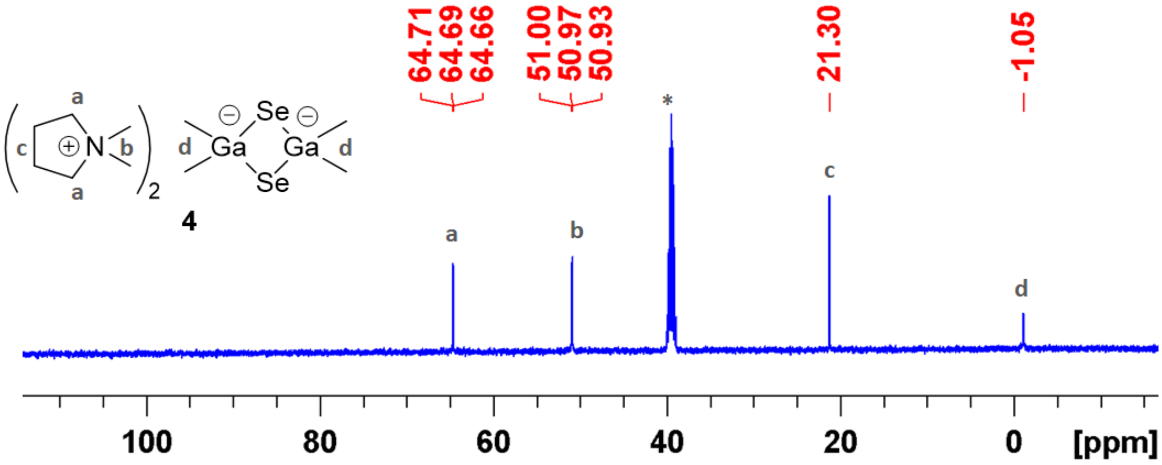

^{13}C-NMR (125.8 MHz, *dmso-d_6) of DMPyr$_2$[Me$_2$Ga(μ_2-Se)]$_2$ (**4**).

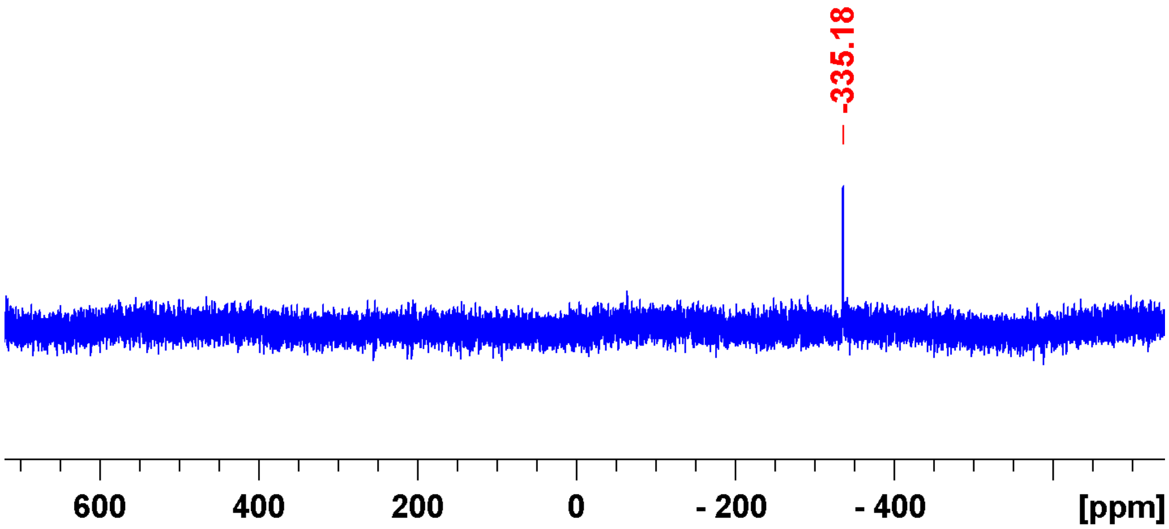

^{77}Se-NMR (57.3 MHz, *dmso-d_6) of DMPyr$_2$[Me$_2$Ga(μ_2-Se)]$_2$ (**4**).

*DMPyr$_2$[Me$_2$In(μ_2-S)]$_2$ (**5**)*

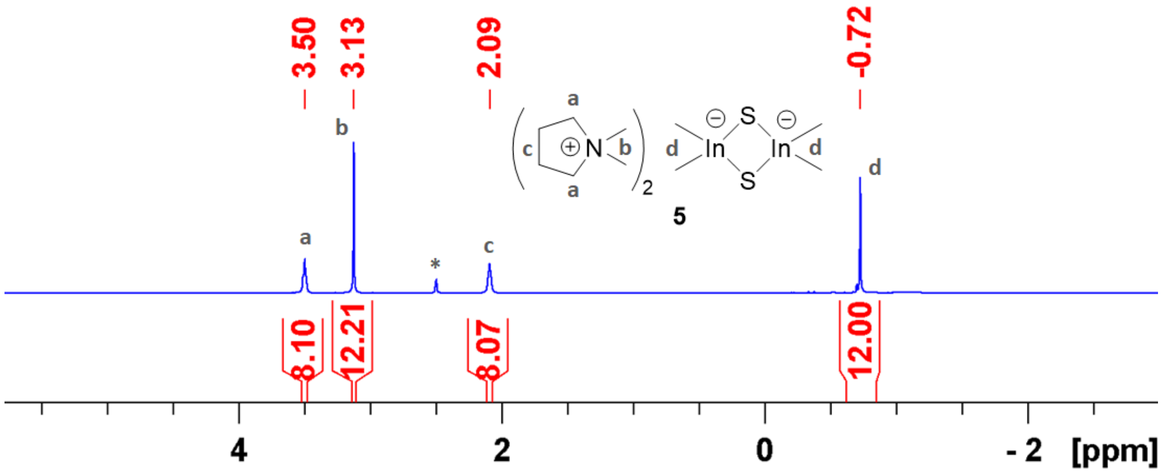

^{1}H-NMR (500.2 MHz, *dmso-d$_6$) DMPyr$_2$[Me$_2$In(μ_2-S)]$_2$ (**5**).

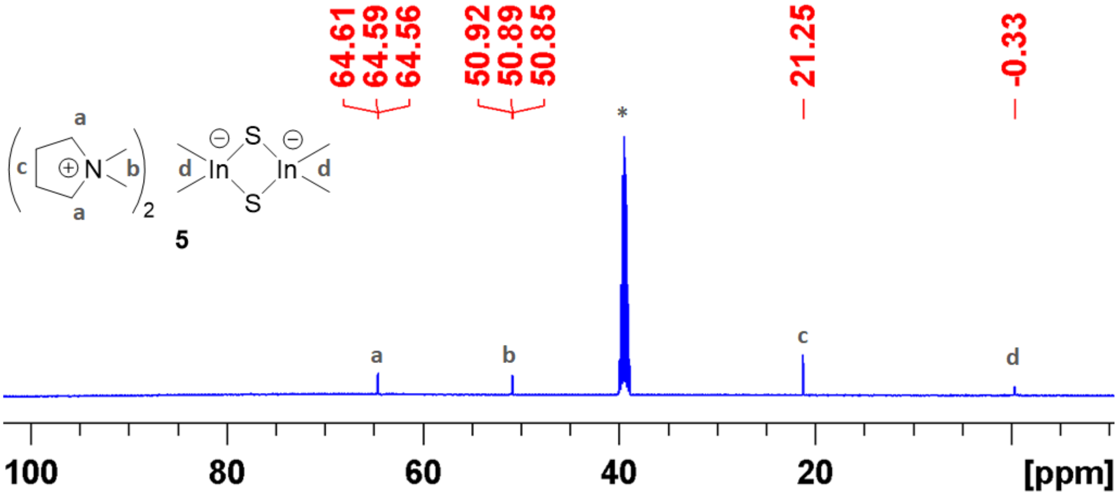

^{13}C-NMR (125.8 MHz, *dmso-d_6) of DMPyr$_2$[Me$_2$In(μ_2-S)]$_2$ (**5**).

*DMPyr$_2$[Me$_2$In(μ_2-Se)]$_2$ (**6**)*

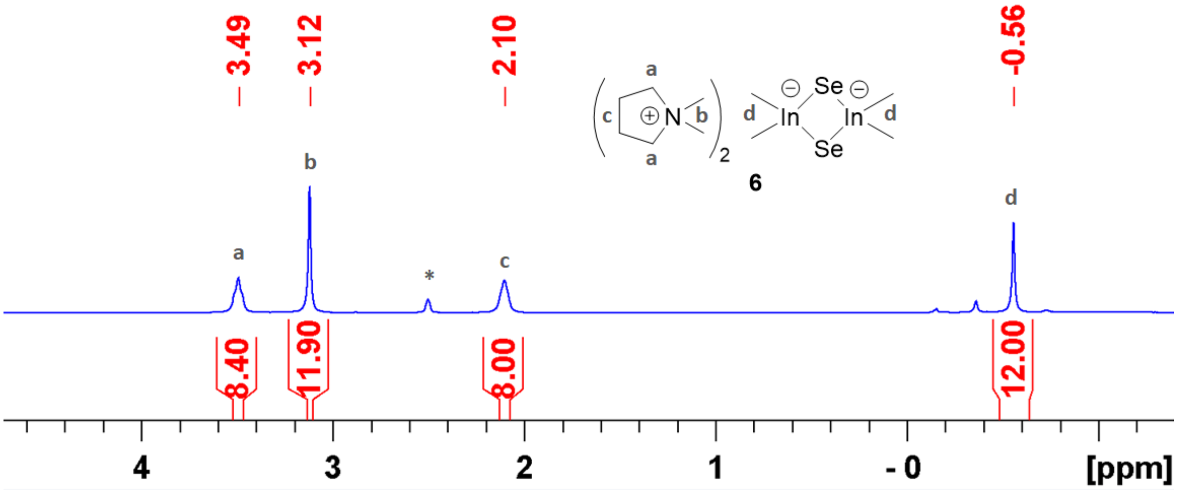

[1]**H-NMR** (300.2 MHz, *dmso-d$_6$) DMPyr$_2$[Me$_2$In(μ_2-Se)]$_2$ (**6**).

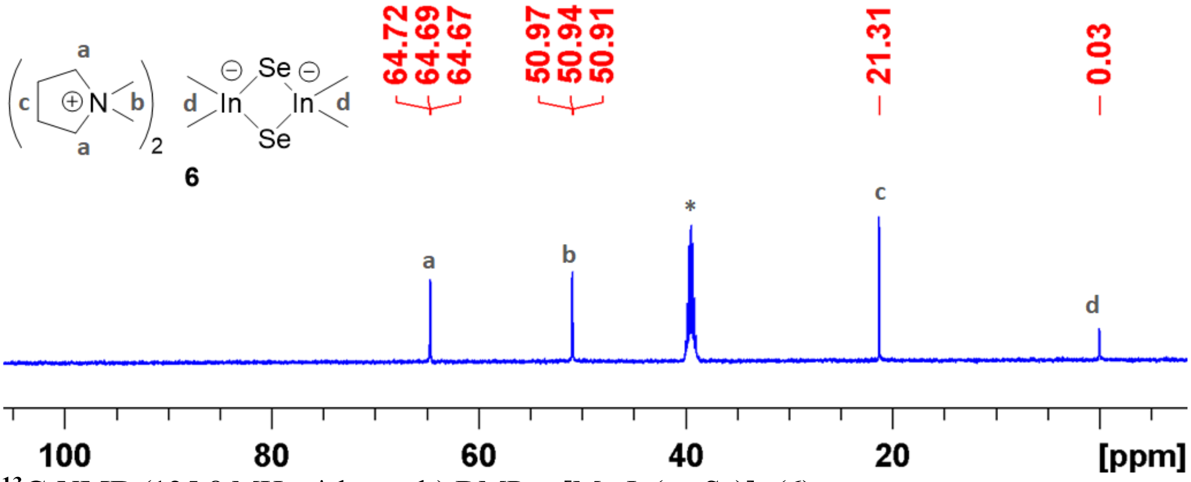

[13]**C-NMR** (125.8 MHz, *dmso-d$_6$) DMPyr$_2$[Me$_2$In(μ_2-Se)]$_2$ (**6**).

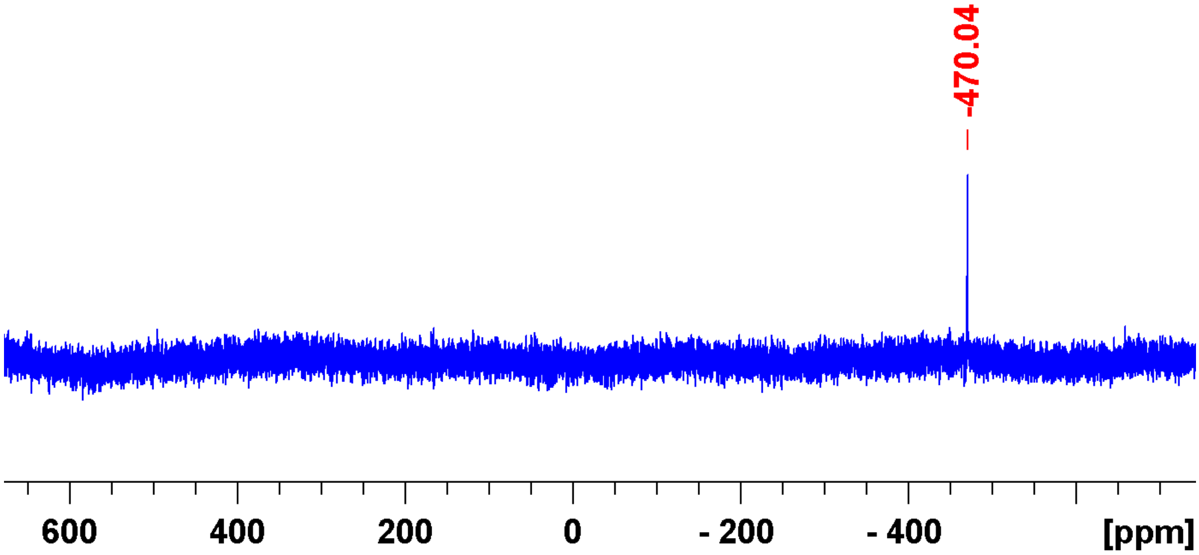

[77]**Se-NMR** (57.3 MHz, *dmso-d$_6$) DMPyr$_2$[Me$_2$In(μ_2-Se)]$_2$ (**6**).

DMPyr[$Me_2In(SSiMe_3)_2$] (7)

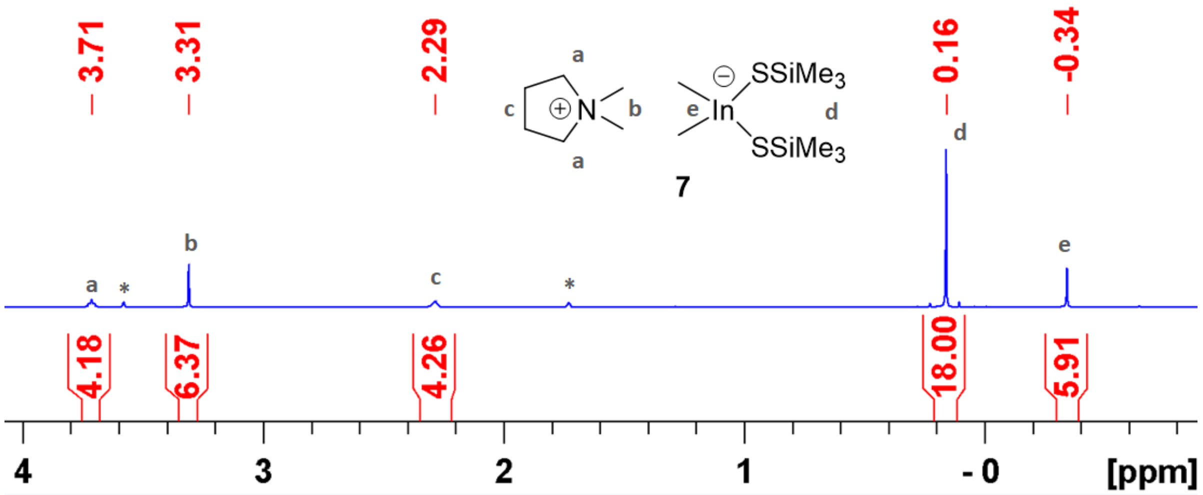

^{1}H-NMR (500.2 MHz, *THF-d_8) of DMPyr [$Me_2In(SSiMe_3)_2$] (**7**)

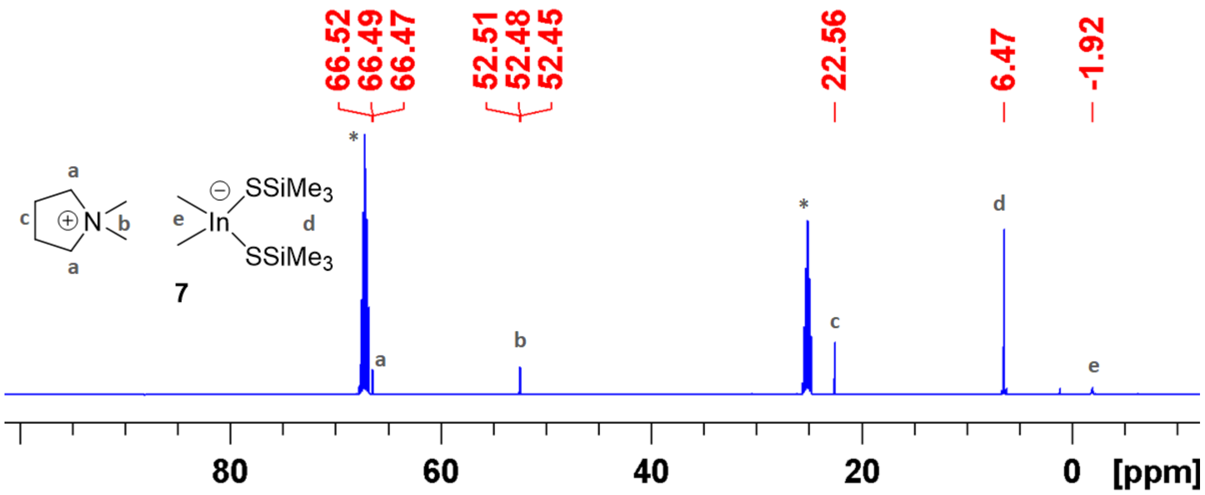

^{13}C-NMR (125.8 MHz, *THF-d_8) of DMPyr [$Me_2In(SSiMe_3)_2$] (7)

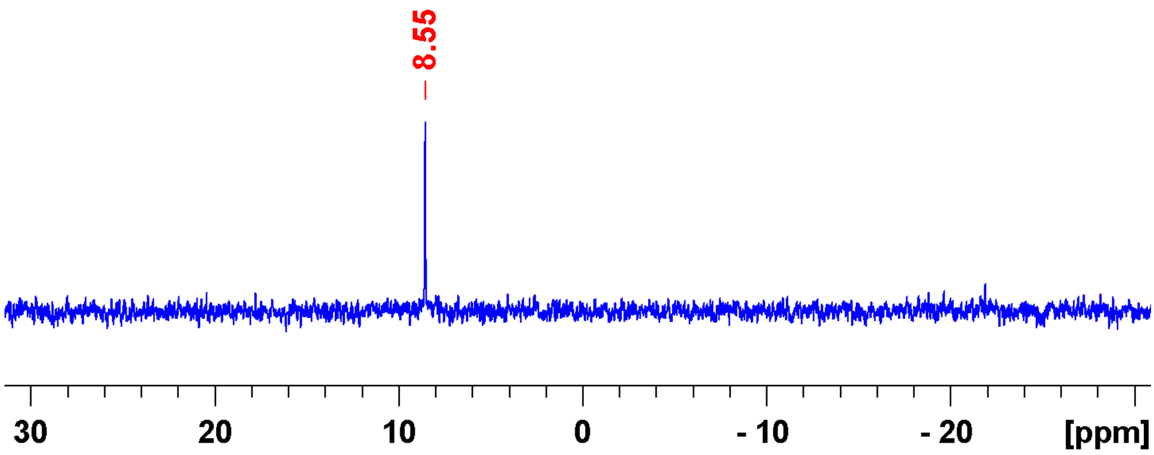

^{29}Si-NMR (99.4 MHz, *THF-d_8) of DMPyr [$Me_2In(SSiMe_3)_2$] (**7**)

*DMPyr[Me$_2$In(SeSiMe$_3$)$_2$] (**8**)*

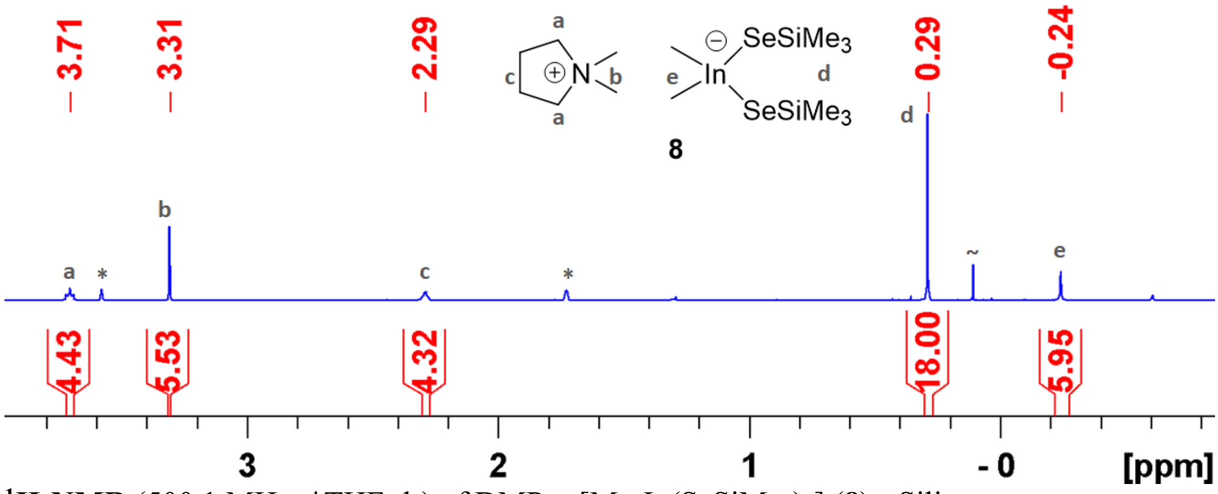

[1]**H-NMR** (500.1 MHz, *THF-d$_8$) of DMPyr [Me$_2$In(SeSiMe$_3$)$_2$] (**8**), ~Silicon grease.

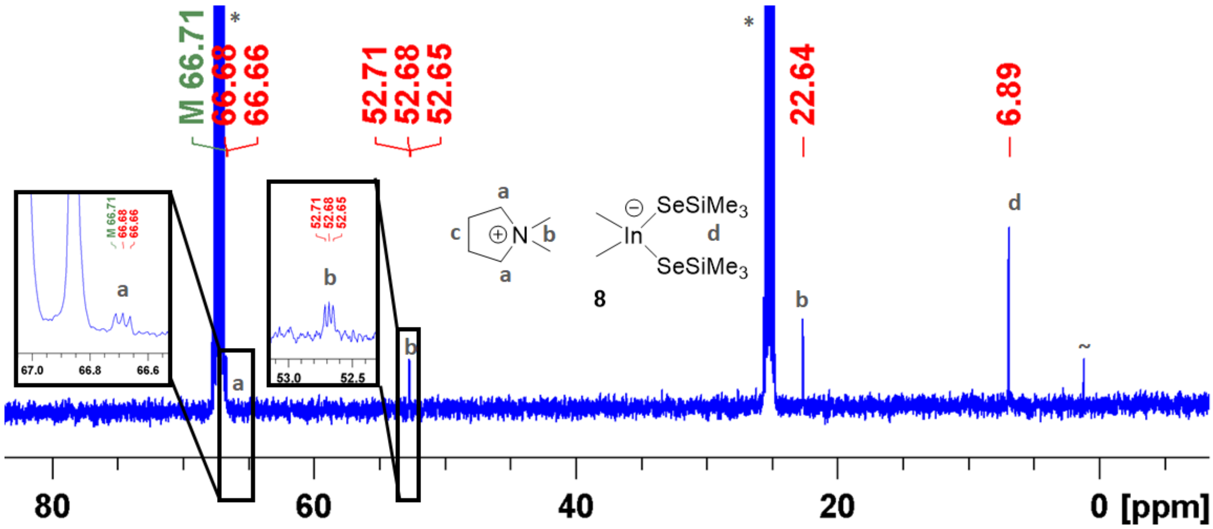

[13]**C-NMR** (125.8 MHz, *THF-d$_8$) of DMPyr [Me$_2$In(SeSiMe$_3$)$_2$] (**8**), ~Silicon grease.

The signal for the indium attached methyl groups could not be identified, as the concentration of the saturated solution of **8** in THF-d$_8$ is not high enough. No ^{29}Si- and ^{77}Se-NMR spectra were obtained for this reason.

*DMPyr[Me$_2$In(SeSiMe$_3$)(SSiMe$_3$)] (**9**)*

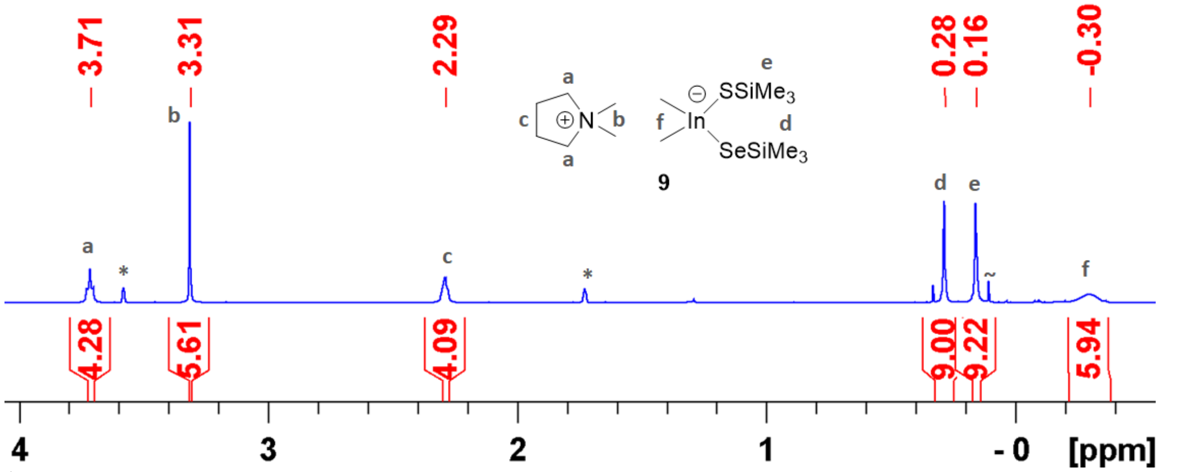

^{1}**H-NMR** (500.1 MHz, *THF-d$_8$) of DMPyr [Me$_2$In(SeSiMe$_3$)(SSiMe$_3$)] (**9**), ~Silicon grease.

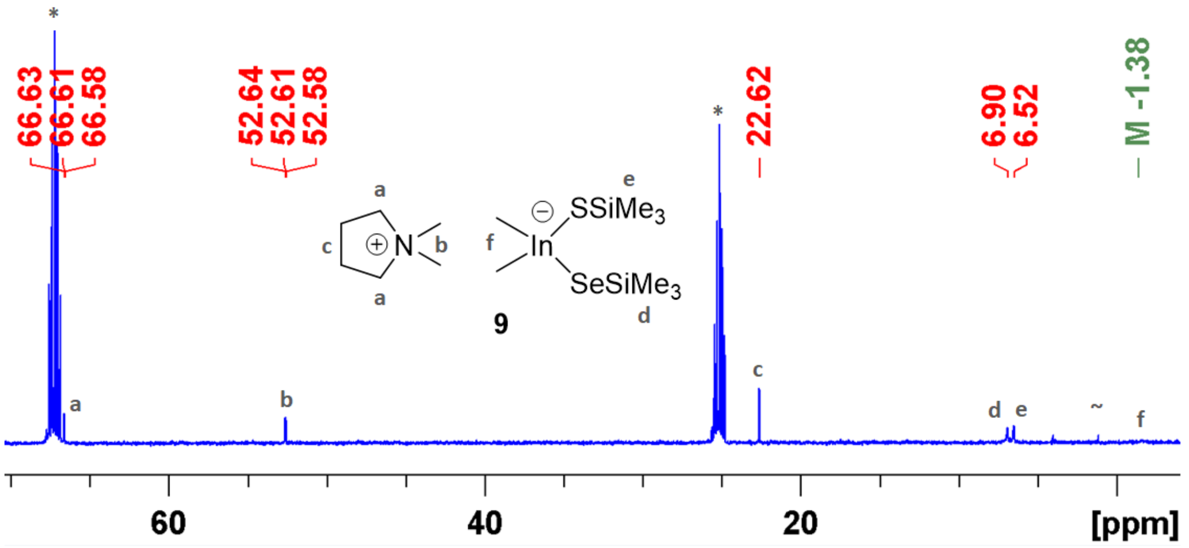

^{13}**C-NMR** (125.8 MHz, *THF-d$_8$) of DMPyr [Me$_2$In(SeSiMe$_3$)(SSiMe$_3$)] (**9**), ~Silicon grease.

The signal for the indium attached methyl groups could hardly be identified, due to the low concentration of the saturated solution of **9** in THF-d$_8$. Therefore no ^{29}Si- and no ^{77}Se-NMR spectra could be obtained.

*DMPyr$_3$[Me$_2$In(μ_2-S-InMe$_3$)]$_3$ (**10**)*

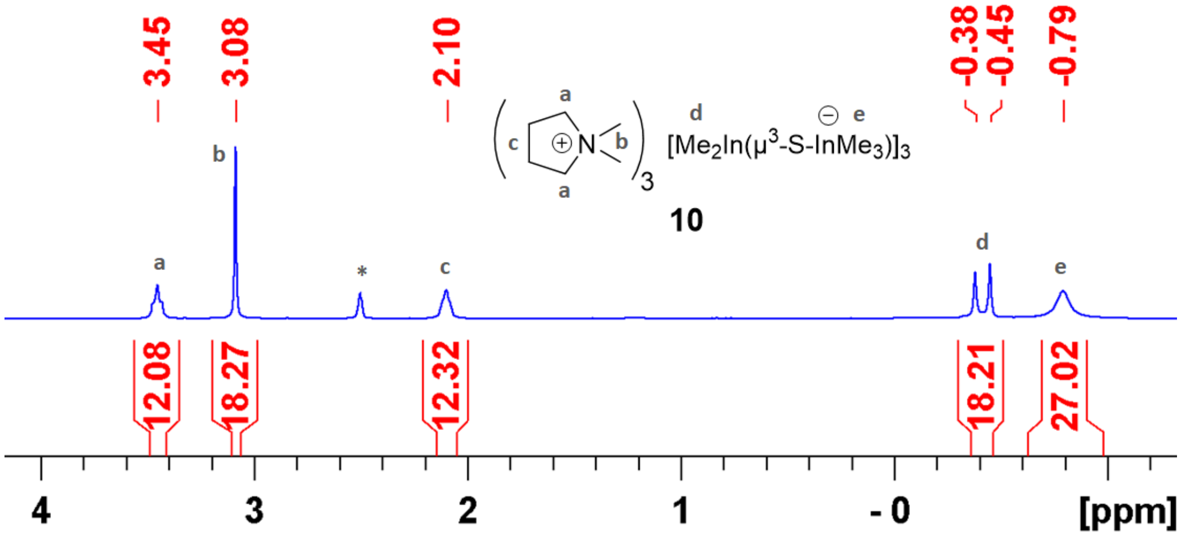

[1]**H-NMR** (300.1 MHz, dmso-d$_6$) of DMPyr$_3$[Me$_2$In(μ_2-S-InMe$_3$)]$_3$ (**10**).

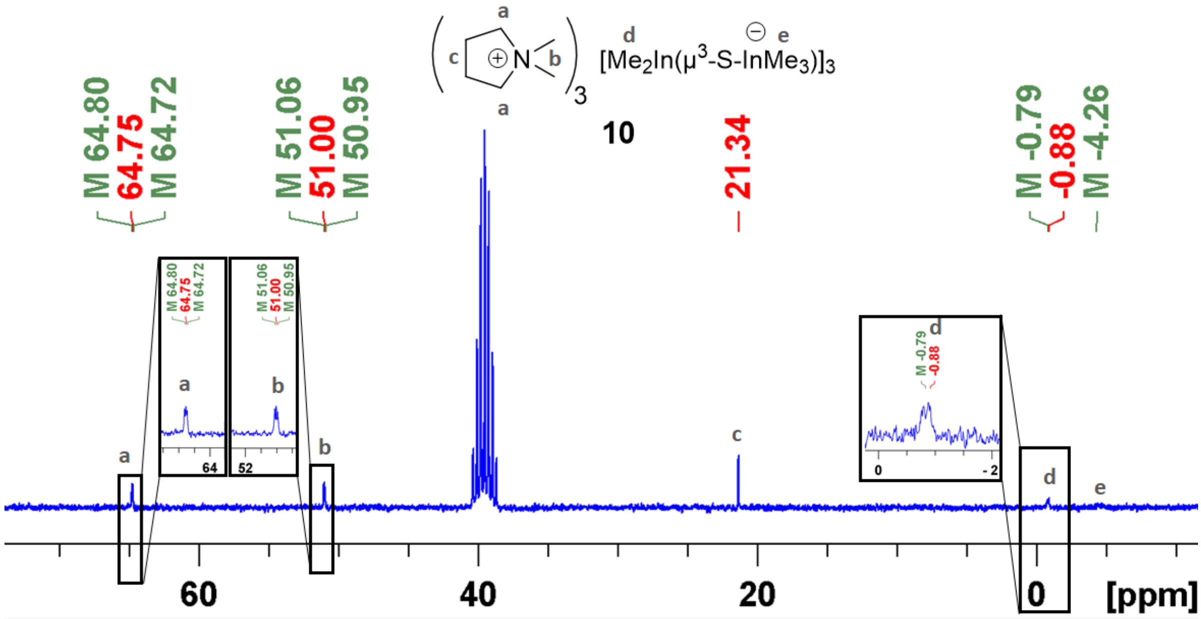

[13]**C-NMR** (75.5 MHz, dmso-d$_6$) of DMPyr$_3$[Me$_2$In(μ_2-S-InMe$_3$)]$_3$ (**10**).

Note that the split signal of the indium attached methyl groups of **10** could be caused by the conformative rigidity determined by the twist-boat conformation that could be identified in the crystallographically determined molecular structure of the anion.

Crystallographic information

The data collection for the single-crystal structure determination was performed on a Stoe Stadivari diffractometer or a Bruker D8 Quest diffractometer by the X-ray service department of the Fachbereich Chemie, University of Marburg. Information concerning the used hard-ware, and software used for Data collection, cell refinement and data reduction as well as structure solution and refinement can be reviewed in the attached CIF-files. After the solution (Shelxt)[5] and refinement process (Shelxl 2017/1)[6] the data was validated by using Platon.[7] All graphic representations were created with Diamond 4.[8]

*XRD data for the hydrochalcogenides DMPyr[SH] (**1**) and DMPyr[SeH] (**2**)*

Table S1: XRD crystal and refinement data of DMPyr[SH] (**1**) and DMPyr[SeH] (**2**).

	**DMPyr[SH] (1)**		**DMPyr[SeH] (2)**	
**CCDC code**	1910798		1910797	
**Identification code**	majg06		jgdmpsehloesen	
**Empirical formula**	C6 H15 N S		C6 H14 N Se	
**Formula weight**	133.25		179.14	
**Temperature**	100(2) K		100(2) K	
**Wavelength**	0.71073 Å		0.71073 Å	
**Crystal system**	Monoclinic		Monoclinic	
**Space group**	$P2_1/n$		$P2_1/n$	
**Unit cell dimensions**	a = 6.7084(4) Å	α= 90°.	a = 6.9056(3) Å	α= 90°.
	b = 11.7617(7) Å	β= 94.632(2)°.	b = 11.8466(6) Å	β= 94.578(4)°.
	c = 9.9595(5) Å	γ = 90°.	c = 10.1507(4) Å	γ = 90°.
**Volume**	783.26(8) $Å^3$		827.76(6) $Å^3$	
**Z**	4		4	
**Density (calculated)**	1.130 Mg/m^3		1.437 Mg/m^3	
**Absorption coefficient**	0.322 mm^{-1}		4.449 mm^{-1}	
**F(000)**	296		364	
**Crystal size**	0.250 x 0.190 x 0.130 mm^3		0.375 x 0.210 x 0.119 mm^3	
**Theta range for data collection**	2.685 to 27.127°.		2.647 to 34.584°.	
**Index ranges**	-8<=h<=7, -14<=k<=15, -12<=l<=12		-10<=h<=10, -18<=k<=14, -16<=l<=14	
**Reflections collected**	8889		14464	
**Independent reflections**	1733 [R(int) = 0.0254]		3345 [R(int) = 0.0670]	
**Completeness to theta = 25.242°**	100.0 %		99.7 %	
**Absorption correction**	Semi-empirical from equivalents		Semi-empirical from equivalents	
**Max. and min. transmission**	0.7455 and 0.7044		0.0926 and 0.0109	
**Refinement method**	Full-matrix least-squares on F2		Full-matrix least-squares on F2	
**Data / restraints / parameters**	1733 / 0 / 79		3345 / 0 / 75	
**Goodness-of-fit on F2**	1.081		1.059	
**Final R indices [I>2sigma(I)]**	R1 = 0.0266, wR2 = 0.0655		R1 = 0.0483, wR2 = 0.1065	
**R indices (all data)**	R1 = 0.0307, wR2 = 0.0674		R1 = 0.0855, wR2 = 0.1191	
**Extinction coefficient**	n/a		n/a	
**Largest diff. peak and hole**	0.252 and -0.196 $e.Å^{-3}$		0.880 and -1.476 $e.Å^{-3}$	

*XRD data for the gallates $DMPyr_2[Me_2Ga(\mu_2\text{-}S)]_2$ (**3**) and $DMPyr_2[Me_2Ga(\mu_2\text{-}Se)]_2$ (**4**)*

Table S2: XRD crystal and refinement data of $DMPyr_2[Me_2Ga(\mu_2\text{-}S)]_2$ (**3**) and $DMPyr_2[Me_2Ga(\mu_2\text{-}Se)]_2$ (**4**).

	$DMPyr_2[Me_2Ga(\mu_2\text{-}S)]_2$ (3)		$DMPyr_2[Me_2Ga(\mu_2\text{-}Se)]_2$ (4)	
**CCDC code**	1910795		1910800	
**Identification code**	majg12C4		jg370loesen	
**Empirical formula**	C16 H40 Ga2 N2 S2		C16 H40 Ga2 N2 Se2	
**Formula weight**	464.06		557.86	
**Temperature**	100(2) K		100(2) K	
**Wavelength**	0.71073 Å		1.54186 Å	
**Crystal system**	Monoclinic		Monoclinic	
**Space group**	$P2_1/n$		$P2_1/n$	
**Unit cell dimensions**	a = 9.5961(4) Å	α= 90°.	a = 9.8332(7) Å	α= 90°.
	b = 12.0450(5) Å	β= 102.3790(10)°.	b = 12.1082(11) Å	β= 102.272(6)°.
	c = 9.9485(4) Å	γ = 90°.	c = 9.9276(8) Å	γ = 90°.
**Volume**	1123.16(8) $Å^3$		1154.99(16) $Å^3$	
**Z**	2		2	
**Density (calculated)**	1.372 Mg/m^3		1.604 Mg/m^3	
**Absorption coefficient**	2.584 mm^{-1}		6.440 mm^{-1}	
**F(000)**	488		560	
**Crystal size**	0.450 x 0.230 x 0.180 mm^3		0.382 x 0.161 x 0.042 mm^3	
**Theta range for data collection**	2.676 to 27.151°.		5.751 to 66.594°.	
**Index ranges**	-12<=h<=12, -15<=k<=15, -12<=l<=12		-11<=h<=11, -14<=k<=13, -7<=l<=11	
**Reflections collected**	37739		11710	
**Independent reflections**	2483 [R(int) = 0.0382]		2042 [R(int) = 0.0997]	
**Completeness to theta = 25.242°**	99.9 %		99.8 %	
**Absorption correction**	Semi-empirical from equivalents		Semi-empirical from equivalents	
**Max. and min. transmission**	0.7455 and 0.5293		0.0600 and 0.0074	
**Refinement method**	Full-matrix least-squares on F2		Full-matrix least-squares on F2	
**Data / restraints / parameters**	2483 / 85 / 170		2042 / 0 / 104	
**Goodness-of-fit on F2**	1.055		0.996	
**Final R indices [I>2sigma(I)]**	R1 = 0.0192, wR2 = 0.0505		R1 = 0.0806, wR2 = 0.1973	
**R indices (all data)**	R1 = 0.0216, wR2 = 0.0513		R1 = 0.0933, wR2 = 0.2072	
**Extinction coefficient**	n/a		n/a	
**Largest diff. peak and hole**	0.591 and -0.257 e.$Å^{-3}$		2.409 and -1.596 e.$Å^{-3}$	

*XRD data for the indates DMPyr$_2$[Me$_2$In(μ_2-S)]$_2$ (**5**) and DMPyr$_2$[Me$_2$In(μ_2-Se)]$_2$ (**6**)*

Table S3: XRD crystal and refinement data of DMPyr$_2$[Me$_2$In(μ_2-S)]$_2$ (**5**) and DMPyr$_2$[Me$_2$In(μ_2-Se)]$_2$ (**6**).

	**DMPyr$_2$[Me$_2$In(μ_2-S)]$_2$ (5)**		**DMPyr$_2$[Me$_2$In(μ_2-Se)]$_2$ (6)**	
**CCDC code**	1910796		1910794	
**Identification code**	majg12c1		jg14	
**Empirical formula**	C16 H40 In2 N2 S2		C16 H40 In2 N2 Se2	
**Formula weight**	554.26		648.06	
**Temperature**	100(2) K		100(2) K	
**Wavelength**	0.71073 Å		0.71073 Å	
**Crystal system**	Monoclinic		Monoclinic	
**Space group**	$P2_1/n$		$P2_1/n$	
**Unit cell dimensions**	a = 9.7075(4) Å	α= 90°.	a = 9.9187(6) Å	α= 90°.
	b = 12.1404(5) Å	β= 100.208(2)°.	b = 12.2925(9) Å	β= 100.434(2)°.
	c = 9.9277(5) Å	γ = 90°.	c = 9.9398(8) Å	γ = 90°.
**Volume**	1151.49(9) Å^3		1191.88(15) Å^3	
**Z**	2		2	
**Density (calculated)**	1.599 Mg/m^3		1.806 Mg/m^3	
**Absorption coefficient**	2.184 mm^{-1}		4.988 mm^{-1}	
**F(000)**	560		632	
**Crystal size**	0.530 x 0.070 x 0.060 mm^3		0.250 x 0.106 x 0.083 mm^3	
**Theta range for data collection**	2.676 to 27.169°.		2.662 to 25.245°.	
**Index ranges**	-12<=h<=12, -15<=k<=15, -12<=l<=12		-11<=h<=11, -14<=k<=14, -11<=l<=11	
**Reflections collected**	34138		11756	
**Independent reflections**	2560 [R(int) = 0.0527]		2148 [R(int) = 0.0464]	
**Completeness to theta = 25.242°**	100.0 %		100.0 %	
**Absorption correction**	Semi-empirical from equivalents		Semi-empirical from equivalents	
**Max. and min. transmission**	0.7455 and 0.6218		0.7452 and 0.6075	
**Refinement method**	Full-matrix least-squares on F2		Full-matrix least-squares on F2	
**Data / restraints / parameters**	2560 / 0 / 104		2148 / 0 / 104	
**Goodness-of-fit on F2**	1.071		1.068	
**Final R indices [I>2sigma(I)]**	R1 = 0.0141, wR2 = 0.0296		R1 = 0.0209, wR2 = 0.0407	
**R indices (all data)**	R1 = 0.0184, wR2 = 0.0303		R1 = 0.0306, wR2 = 0.0431	
**Extinction coefficient**	n/a		n/a	
**Largest diff. peak and hole**	0.277 and -0.367 e.Å^{-3}		0.508 and -0.382 e.Å^{-3}	

XRD data for the inverse heteroadamantane cage compound DMPyr$_2$[(Me$_2$In)$_6$(μ_3-S)$_4$] ***(11)***

Table S4: XRD crystal and refinement data of DMPyr$_2$[(Me$_2$In)$_6$(μ_3-S)$_4$] (**11**).

	DMPyr$_2$[(Me$_2$In)$_6$(μ_3-S)$_4$] (11)	
**CCDC code**	1910799	
**Identification code**	majgl2inb	
**Empirical formula**	C28 H72 In6 N2 O1 S4	
**Formula weight**	1270.03	
**Temperature**	100(2) K	
**Wavelength**	0.71073 Å	
**Crystal system**	Tetragonal	
**Space group**	$I4_1/a\ c\ d$	
**Unit cell dimensions**	a = 17.0977(11) Å	α= 90°.
	b = 17.0977(11) Å	β= 90°.
	c = 32.377(2) Å	γ = 90°.
**Volume**	9464.7(14) Å^3	
**Z**	8	
**Density (calculated)**	1.783 Mg/m^3	
**Absorption coefficient**	3.072 mm^{-1}	
**F(000)**	4960	
**Crystal size**	0.435 x 0.403 x 0.195 mm^3	
**Theta range for data collection**	2.382 to 27.143°.	
**Index ranges**	-21<=h<=17, -19<=k<=21, -41<=l<=41	
**Reflections collected**	40556	
**Independent reflections**	2619 [R(int) = 0.0961]	
**Completeness to theta = 25.242°**	99.9 %	
**Absorption correction**	Semi-empirical from equivalents	
**Max. and min. transmission**	0.7455 and 0.4882	
**Refinement method**	Full-matrix least-squares on F2	
**Data / restraints / parameters**	2619 / 132 / 131	
**Goodness-of-fit on F2**	1.037	
**Final R indices [I>2sigma(I)]**	R1 = 0.0377, wR2 = 0.0606	
**R indices (all data)**	R1 = 0.0676, wR2 = 0.0678	
**Extinction coefficient**	n/a	
**Largest diff. peak and hole**	0.878 and -0.689 e.Å^{-3}	

*Structural XRD consideration for DMPyr$_3$[Me$_2$In(μ_2-S-InMe$_3$)]$_3$ (**10**)*

Data was collected with a Bruker D8 QUEST area detector diffractometer equipped with with MoK$_\alpha$ radiation, a graded multilayer mirror monochromator (λ = 0.71073 Å) and a PHOTON-100 CMOS detector using an oil-coated shock-cooled crystal at 100(2) K. Absorption effects were corrected semi-empirical using multiscanned reflexions (SAINT V8.37A (Bruker AXS Inc., 2015)). Cell constants were refined using 9785 of observed reflections of the data collection. The structure was solved by direct methods by using the program XT V2014/1 (Bruker AXS Inc., 2014) and refined by full matrix least squares procedures on F^2 using SHELXL-2017/1 (Sheldrick, 2017). The non-hydrogen atoms have been refined anisotropically, carbon bonded hydrogen atoms were included at calculated positions and refined using the 'riding model' with isotropic temperature factors at 1.2 times (for CH_3 groups 1.5 times) that of the preceding carbon atom. Data were of low quality. Only data up to Theta 23.3 was used during refinement. Split positions were refined for the In and S positions. Large anisotrop displacement factors for the cations indicate non-resolved disorder. High difference electron density indicates that the refinement is not complete.

10 crystallizes in the space group *P*–1 with eight formula units per unit cell. Despite the low quality of the crystallographic data, the shape and the conformation and the connectivity of the anion can be qualitatively identified. A discussion of bond lengths and angles is only possible on a coarse level. The anion has a trimer-like six-membered ring structure in the twist-boat conformation that allows the terminal trimethylindate moieties to obtain an equatorial orientation. As methyl groups attached to the cyclic bond indium atoms obtain an axial and an equatorial orientation, we assume these methyl groups' signals in the ^{1}H- and ^{13}C-NMR-spectra are split into two. In **figure S1** a qualitatively representative anion from the eight different formula units set of XRD data is shown without disordered atoms.

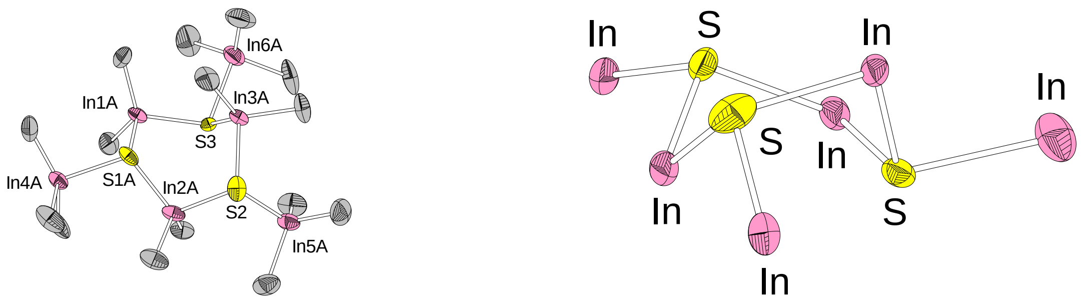

Figure S1: Crystallographically determined molecular structure of the anion present in DMPyr$_3$[(Me$_2$In(μ_3-S-InMe$_3$)]$_3$ (**10**) (left hand side: trianion) with a twist-boat conformation (right hand side: binary framework of the anion). Protons, disordered atoms, and cations are not shown for clarity. Only one of eight crystallographically independend anions of the elemental cell is shown. Ellipsoids shown in 50% level. Selected bond lengths (in Å) and angles (°) of the shown representative anion: In1A-S1A 2.51(2); S1A-In2A 2.51(2); In2A-S2 2.57(3); S2-In3A 2.64(2); In3A-S3 2.49(2); S3-In1A 2.50(2); S1A-In4A 2.57(3); S1A-In4A 2.57(3); S2-In5A 2.44(2); S3-In6A 2.66(2), S1A-In2A-S2 102.6(7), In2A-S2-In3A 100.1(8), S2-In3A-S3 103.3(7), In3A-S3-In1A 105.2(7), S3-In1A-S1A 99.9(7), In1A-S1A-In2A 105.8(6), In1A-S1A-In2A-In4A –109.8(9), In2A-S2-In3A-In5A –108(1), In3A-S3-In1A-In6A 98.0(9), S1A-In1A-S3-In3A –44.6(8), S3-In3A-S2-In2A –32.8(9).

Table S5: XRD crystal and refinement data for $DMPyr_3[Me_2In(\mu_2\text{-S-InMe}_3)]_3$ (**10**).

	**$DMPyr_3[(Me_2In(\mu_2\text{-S-InMe}_3)]_3$ (10)**	
Identification code	JG085_0m	
Habitus, colour	colorless needle	
Crystal size	0.180 x 0.170 x 0.120 mm^3	
Crystal system	Triclinic	
Space group	P-1	Z = 8
Unit cell dimensions	a = 18.4827(7) Å	α= 61.516(2)°.
	b = 25.5703(11) Å	β= 87.572(2)°.
	c = 26.6454(12) Å	γ = 69.629(2)°.
Volume	10258.7(8) $Å^3$	
Cell determination	9785 peaks with Theta 2.3 to 20.8°.	
Empirical formula	C33 H87 In6 N3 S3	
Moiety formula	C15 H45 In6 S3, 3(C6 H14 N)	
Formula weight	1311.15	
Density (calculated)	1.698 Mg/m^3	
Absorption coefficient	2.797 mm^{-1}	
F(000)	5184	
Data collection:		
Diffractometer type	Bruker D8 QUEST area detector	
Wavelength	0.71073 Å	
Temperature	100(2) K	
Theta range for data collection	2.208 to 23.257°.	
Index ranges	-20<=h<=20, -28<=k<=28, -29<=l<=29	
Data collection software	APEX3 (Bruker AXS Inc., 2015)[9]	
Cell refinement software	SAINT V8.37A (Bruker AXS Inc., 2015)[10]	
Data reduction software	SAINT V8.37A (Bruker AXS Inc., 2015)	
Solution and refinement:		
Reflections collected	124803	
Independent reflections	29472 [R(int) = 0.0549]	
Completeness to theta = 23.257°	99.9 %	
Observed reflections	21271[I > 2σ(I)]	
Reflections used for refinement	29472	
Absorption correction	Semi-empirical from equivalents[11]	
Max. and min. transmission	0.2049 and 0.1669	
Largest diff. peak and hole	3.046 and -1.518 e.$Å^{-3}$	
Solution	dual/ difmap[12,13]	
Refinement	Full-matrix least-squares on F2[13]	
Treatment of hydrogen atoms	mixed, constr	
Programs used	XT V2014/1 (Bruker AXS Inc., 2014),<br>SHELXL-2017/1 (Sheldrick, 2017),<br>DIAMOND (Crystal Impact),<br>ShelXle (Hübschle, Sheldrick, Dittrich, 2011)	
Data / restraints / parameters	29472 / 5054 / 1949	
Goodness-of-fit on F2	1.078	
R index (all data)	wR2 = 0.2350	
R index conventional [I>2sigma(I)]	R1 = 0.0911	

Alternative attempts to synthesize DMPyr$_2$[(Me$_2$In)$_6$(μ$_3$-S)$_4$] (11) on gram scale

As the investigated single crystal that could be crystallographically identified as DMPyr$_2$[(Me$_2$In)$_6$(μ_3-S)$_4$] (**11**) emerged from a sample that contained a saturated solution of DMPyr$_3$[(Me$_2$In)(μ_2-S-InMe$_3$)]$_3$ (**10**) in tetrahydrofurane and was stored at room temperature for several weeks, we assumed **11** to be a thermal decomposition product of **10**. To support this theory some experiments were conducted: the defined thermolysis of **10**, and several attempts to synthesize the inverse heteroadamantane cage anion.

*Thermolysis of **10***

One possible access to **11** is the thermal treatment of **10**, as it is possible to formulate a dismutation mechanism in which four equivalents of **10** decompose to six equivalents of DMPyr[InMe$_4$] and three equivalents of **11** (**Scheme S1**).

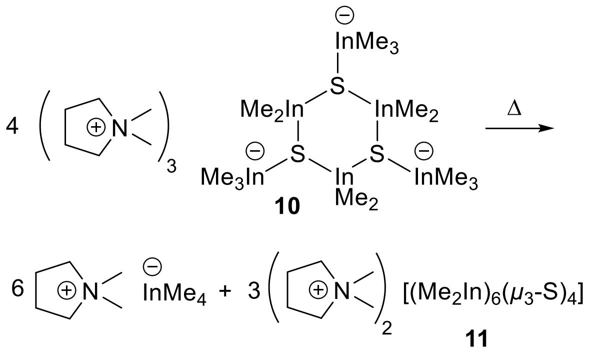

Scheme S1. Proposed dismutation mechanism for the formation of **11** by thermolysis of **10**.

To support this assumption a defined thermolysis experiment was conducted. As the solubility of DMPyr[InMe$_4$] and **11** are comparable, the emerging mixture is investigated.

A solution of 50 mg of **10** in diglyme (10 mL) was stirred at 100 °C for 18 h. All volatiles were removed in fine vacuum and the remaining colorless solid is dried in fine vacuum. The ^{1}H-NMR spectrum fits a mixture of six equivalents of DMPyr[InMe$_4$] and three equivalents of **11**.

^{1}H-NMR (300.1 MHz, dmso-d$_6$): δ_H = 3.45 (m, 48H, 9 x (H$_2$C)$_2$(H_2C)$_2$N(CH$_3$)$_2$), 3.08 (s, 63H, 9 x (H$_2$C)$_2$(H$_2$C)$_2$N(CH_3)$_2$), 2.08 (m, 48H, 9 x (H$_2$C)$_2$(H$_2$C)$_2$N(CH_3)$_2$), –0.38 (bs, 72H, 6 x In(CH$_3$)$_4$), –0.64 (bs, 108H, 3 x [(H_3C)$_2$In)$_6$(μ_3-S)$_4$]) ppm. We point out, that the reliability of integration is restricted, as the signals are quite broad (**figure S2**).

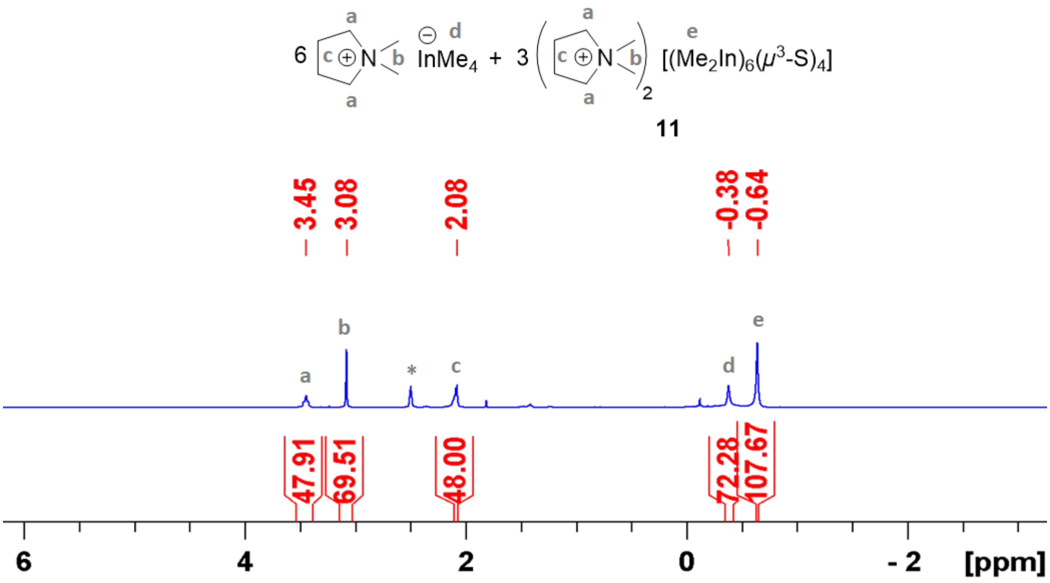

Figure S2: **^{1}H-NMR** (300.1 MHz, *dmso-d$_6$) of the thermolysis product of **10**.

To realize the preparation of **11**, we tried to perform the formation of the inverse heteroadamantane cage by the reactions shown in **Scheme S3**: via elimination of $SiMe_4$ (**Scheme S2**, A), or $ClSiMe_3$ (**Scheme S2**, B/C). These reaction pathways were chosen to avoid nonvolatile byproducts. However, it was not possible to prepare **11** (or the representative with Ph_4P^+ cation **11-Ph$_4$P**) purely.

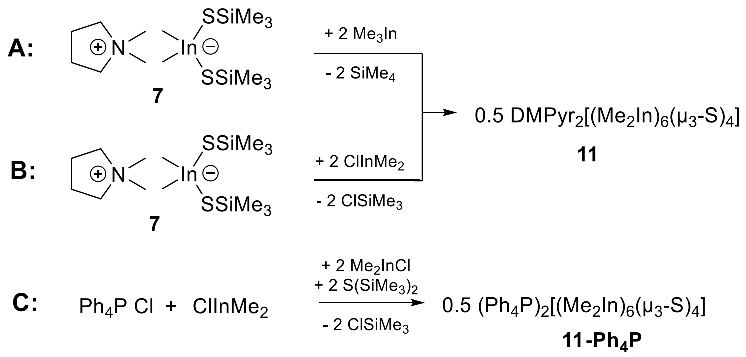

Scheme S2. Attempts to prepare **11** by elimination of $SiMe_4$ (**A**) or $ClSiMe_3$ (**B**/**C**).

*A: Attempt to prepare **11** starting from 7 by elimination of SiMe$_4$*

To a solution of DMPyr[$Me_2In(SSiMe_3)_2$] (**7**) (0.076 g, 0.16 mmol, 2.0 eq.) in 5 mL thf a solution of Me_3In (0.053 g, 0.32 mmol, 4.0 eq.) in 5 mL thf was added dropwise at –100 °C. The reaction mixture was allowed to obtain room temperature during a time period of 18 h. All volatiles of the

slightly cloudy mixture were removed in fine vacuum. The colorless residue was dried in fine vacuum and investigated by ^{1}H-NMR spectroscopy.

^{1}H-NMR (300.1 MHz, dmso-d$_6$): δ_H = 3.44 (m, 8H, 2 x $(H_2C)_2(H_2C)_2N(CH_3)_2$), 3.08 (s, 12H, 12 x $(H_2C)_2(H_2C)_2N(CH_3)_2$), 2.08 (m, 8H, 2 x $(H_2C)_2(H_2C)_2N(CH_3)_2$), –0.63 (s, 36H, $[(H_3C)_2In)_6(\mu_3$-$S)_4]$) ppm. Impurities are indicated by signals at 0.06 ppm, –0.11 ppm, and –0.36 ppm with a total intensity of 41 proton equivalents per formula unit of the desired product. The impurities cannot be removed by washing with pentane or diethyl ether (**figure S3**). The signal at –0.63 ppm is assumed to be the signal for the $[(H_3C)_2In)_6(\mu_3$-$S)_4]^{2-}$ dianion (compare the signal at –0.64 ppm in **figure S2**).

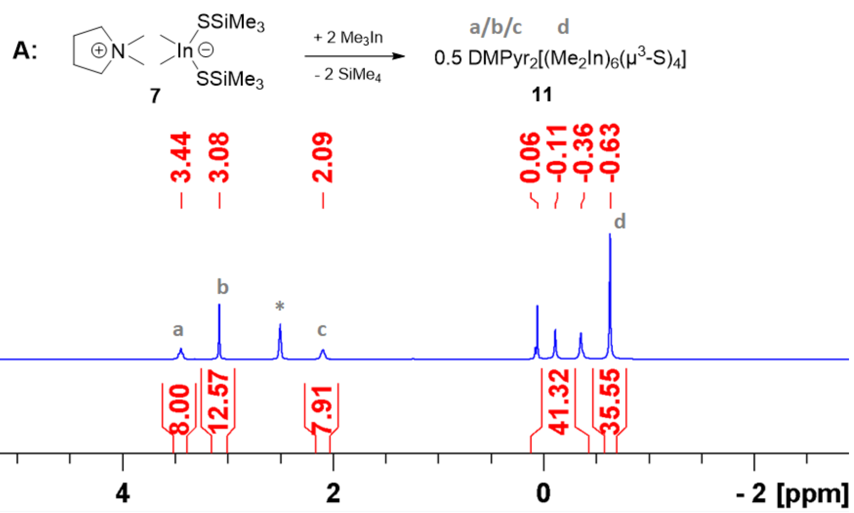

Figure S3: **^{1}H-NMR** (300.1 MHz, *dmso-d$_6$) of the attempt to synthesize compound **11** by elimination of $SiMe_4$. Impurities between 0.06 ppm and –0.36 ppm could not be avoided or removed.

***B**: Attempt to prepare **11** starting from 7 by elimination of $ClSiMe_3$*

DMPyr[$Me_2In(SSiMe_3)_2$] (**7**) (0.050 g, 0.11 mmol, 2.0 eq.) and $ClInMe_2$ (0.043 g, 0.22 mmol, 4.0 eq.) are diluted in in 5 mL thf at –78 °C. The reaction mixture was allowed to obtain room temperature during a time period of 18 h. All volatiles of the slightly cloudy mixture were removed in fine vacuum. The colorless residue was dried in fine vacuum and investigated by ^{1}H-NMR spectroscopy.

^{1}H-NMR (300.1 MHz, dmso-d$_6$): δ_H = 3.45 (m, 8H, 2 x $(H_2C)_2(H_2C)_2N(CH_3)_2$), 3.08 (s, 12H, 12 x $(H_2C)_2(H_2C)_2N(CH_3)_2$), 2.10 (m, 8H, 2 x $(H_2C)_2(H_2C)_2N(CH_3)_2$), –0.63 (s, 6H,* $[(H_3C)_2In)_6(\mu_3$-$S)_4]$) ppm. *The intensity of the signal at –63 ppm is too low indicating a uncomplete conversion. Impurities are indicated by signals at 0.06 ppm, –0.11 ppm, and –0.36 ppm with a total intensity of 36 proton equivalents per formula unit of the desired product. The impurities cannot be removed by washing with pentane or diethyl ether (**Figure S4**).

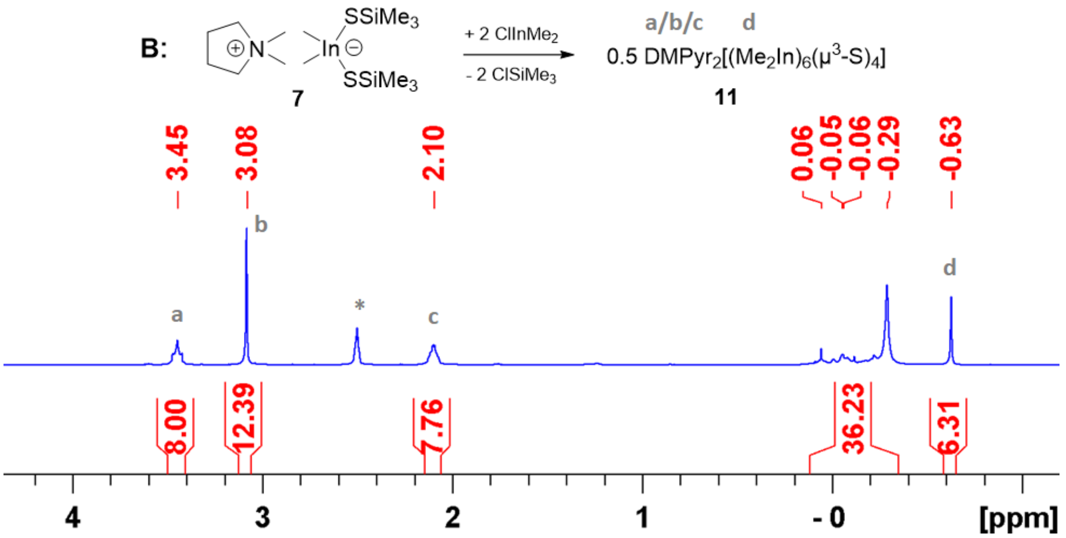

Figure S4: **^{1}H-NMR** (300.1 MHz, *dmso-d$_6$) of the attempt to synthesize compound **11** by elimination of $ClSiMe_3$. Impurities between 0.06 ppm and –0.29 ppm could not be avoided or removed.

*C: Attempt to prepare **11-Ph$_4$P** starting from 7 by elimination of $ClSiMe_3$*

To a suspension of $Ph_4P[Cl]$ (0.067 g, 0.17 mmol, 2.0 eq.) in 5 mL thf a solution of $ClInMe_2$ (0.032 g, 0.17 mmol, 2.0 eq.) in 5 mL thf was stirred for 18 h at room temperature to obtain a clear solution of $Ph_4P[Me_2InCl_2]$. $S(SiMe_3)_2$ (0.064 g, 0.36 mmol, 4.0 eq.) was added dropwise to a solution of Me_2InCl (0.065 g, 0.36 mmol, 4.0 eq.) in 5 mL at –20 °C. The solution was allowed to obtain room temperature within a time period of 18 h to obtain a solution of $[Me_2In(\mu_2\text{-}SSiMe_3)]_2$. To the $Ph_4P[Me_2InCl_2]$ solution the solution of $[Me_2In(\mu_2\text{-}SSiMe_3)]_2$ was slowly added at –78 °C. The reaction mixture was allowed to warm to room temperature within a time period of 18 h. All volatiles of the solution were removed in fine vacuum. The colorless residue was dried in fine vacuum and investigated by ^{1}H-NMR spectroscopy.

^{1}H-NMR (300.1 MHz, dmso-d$_6$): δ_H = 7.97-7.78 (m, 40 H, $(C_6H_5)_4P$), –0.63 (s, 6H*, $[(H_3C)_2In)_6(\mu_3\text{-}S)_4]$) ppm. *The intensity of the signal at –63 ppm is too low, indicating a uncomplete conversion. Impurities are indicated by signals between 0.06 ppm and –0.29 ppm with a total intensity of 36 proton equivalents per two cations. The impurities cannot be removed by washing with pentane or diethyl ether (**Figure S5**).

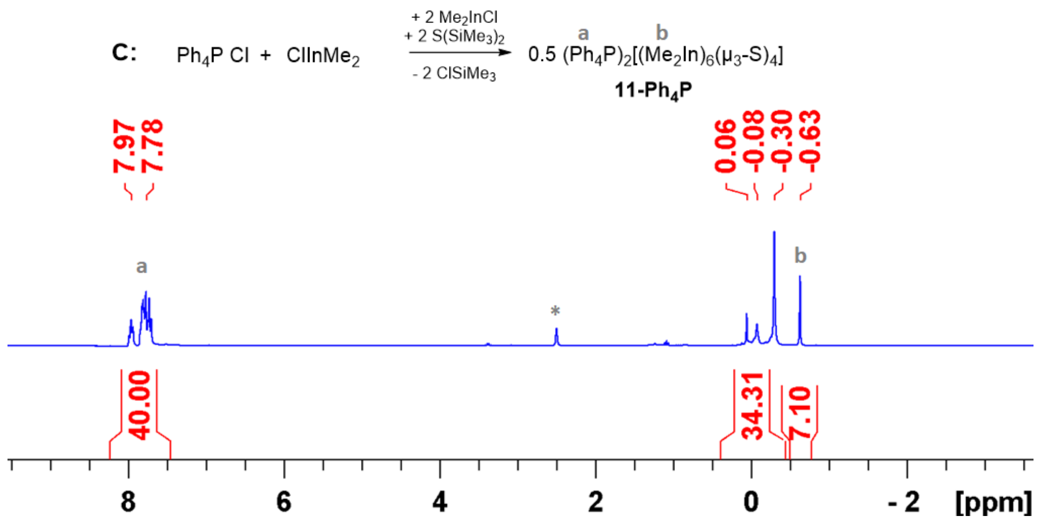

Figure S5: **^{1}H-NMR** (300.1 MHz, *dmso-d$_6$) of the attempt to synthesize compound **11-Ph$_4$P** by elimination of ClSiMe$_3$. Impurities between 0.06 ppm and –0.30 ppm could not be avoided or removed.

In **Figure S6** the ^{1}H-NMR spectra of the attempts to prepare 11 from **Scheme S2**, and the ^{1}H-NMR spectra obtained by the thermolysis of **10** (**Scheme S1**) are layered together. The signal assigned to the inverse heteroadamantane cage anion $[(H_3C)_2In)_6(\mu_3\text{-}S)_4]^{2-}$ at the ^{1}H-NMR spectrum (– 0.64 ppm) of the thermolysis experiment can be found in the attempts to synthesise the anion (–0.63 ppm, the minor deviation could be a concentration effect). We think this is a good reason to assume this signal to be assignable to the $[(H_3C)_2In)_6(\mu_3\text{-}S)_4]^{2-}$ anion. Though we want to point out, that we were not able to perform a complete and selective synthesis of **11**.

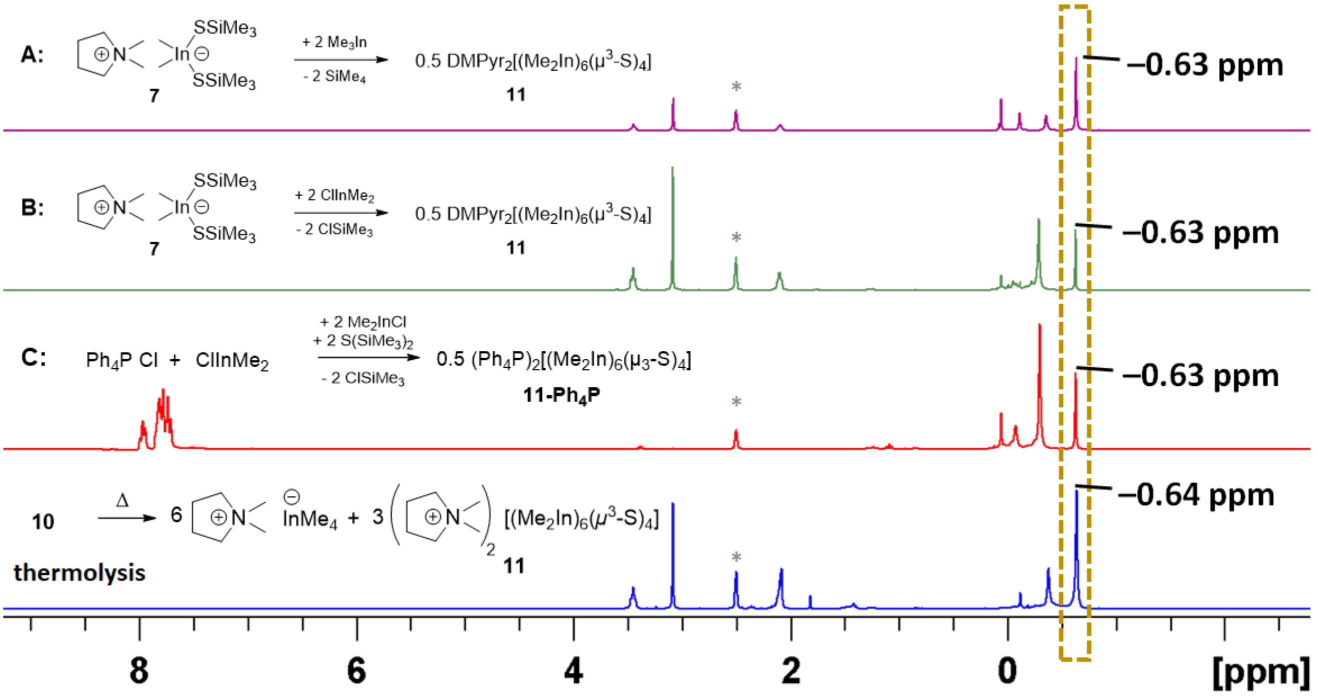

Figure S6: ^{1}H-NMR (300.1 MHz, *dmso-d$_6$) spectra of the thermolysis product of **10** (bottom row) and the attempts to synthesize compounds **11** (**A**/**B**) and **11-Ph$_4$P** (**C**) that contain the $[(H_3C)_2In)_6(\mu_3\text{-}S)_4]^{2-}$ anion.

References

(1) Armarego, W. L. F.; Perrin, D. D. *Purification of laboratory chemicals*, 4. ed., reprint; Butterworth-Heinemann: Oxford, 2002.

(2) Finger, L. H.; Scheibe, B.; Sundermeyer, J. Synthesis of organic (trimethylsilyl)chalcogenolate salts CatTMS-E (E = S, Se, Te): The methylcarbonate anion as a desilylating agent. *Inorg. chem.* **2015**, *54*, 9568–9575, DOI: 10.1021/acs.inorgchem.5b01665.

(3) Brauer, G. *Handbook of Preparative Inorganic Chemistry V2*, 2nd ed.; Elsevier Science: Burlington, 1965.

(4) So, J.-H.; Boudjouk, P. Convenient Syntheses of Hexamethyldisilathiane and Tetramethyldisilathiane. *Synthesis* **1989**, *1989*, 306–307, DOI: 10.1055/s-1989-27235.

(5) Sheldrick, G. M. Crystal structure refinement with SHELXL. *Acta crystallographica. Section C, Structural chemistry* **2015**, *71*, 3–8, DOI: 10.1107/S2053229614024218.

(6) Hübschle, C. B.; Sheldrick, G. M.; Dittrich, B. ShelXle: A Qt graphical user interface for SHELXL. *Journal of applied crystallography* **2011**, *44*, 1281–1284, DOI: 10.1107/S0021889811043202.

(7) Spek, A. L. Structure validation in chemical crystallography. *Acta crystallographica. Section D, Biological crystallography* **2009**, *65*, 148–155, DOI: 10.1107/S090744490804362X.

(8) K. Brandenburg, H. P. *Diamond*; Crystal Impact GbR: Bonn, **2012**.
